# Hybrid biomaterials-based radiosensitizers: Preparations and their applications in enhancing tumor radiotherapy

**DOI:** 10.1016/j.mtbio.2025.102186

**Published:** 2025-08-09

**Authors:** Jia Liu, Lin Zhao, Yang Sun, Qinrui Fu, Wenjing Xiao

**Affiliations:** a Department of Radiotherapy, The Affiliated Hospital of Qingdao University, Qingdao University, Qingdao, 266003, China; b Institute of Chronic Disease, College of Medicine, Qingdao University, Qingdao, 266071, China

**Keywords:** Hybrid biomaterials-based radiosensitizers, Radiotherapy, Reactive oxygen species, Synergistic therapy, Nanomedicine

## Abstract

Radiotherapy is a critical modality in cancer treatment, yet its efficacy can be substantially hindered by the tumor microenvironment. Nanotechnology has empowered nanomaterials to assume multifunctional roles. These roles encompass drug delivery, radiosensitization, imaging, inducing DNA damage, and decreasing glutathione (GSH) levels to impede the neutralization of reactive oxygen species (ROS), thus enhancing therapeutic efficacy. Consequently, hybrid materials integrating multiple functional components have garnered significant attention. In recent years, the development of hybrid biomaterials-based radiosensitizers has successfully addressed these challenges and attracted considerable interest. This review systematically summarizes the synthesis methods, composition, and potential applications of hybrid biomaterials-based radiosensitizers in radiotherapy. The synthesis techniques are classified into self-assembly, chemical synthesis, and biomimetic synthesis. Furthermore, we examine various types of inorganic, inorganic-organic, and organic hybrid biomaterials-based radiosensitizers, with an emphasis on their mechanisms of action, including enhancing ionizing radiation effects, alleviating tumor hypoxia, and depleting GSH. Finally, we discuss the future prospects and existing challenges of hybrid biomaterials-based radiosensitizers in cancer therapy, highlighting their potential to markedly improve therapeutic efficacy.

## Graphical abstract

This review presents the classification and synthesis strategies of hybrid biomaterials-based radiosensitizers, covering inorganic, organic, and inorganic-organic systems. These systems, developed via self-assembly, chemical, and biomimetic methods, integrate multiple functional components into versatile platforms. By enhancing tumor targeting, modulating the tumor microenvironment, and amplifying radiosensitization mechanisms, these platforms improve the effectiveness of radiotherapy.

**Figure:**
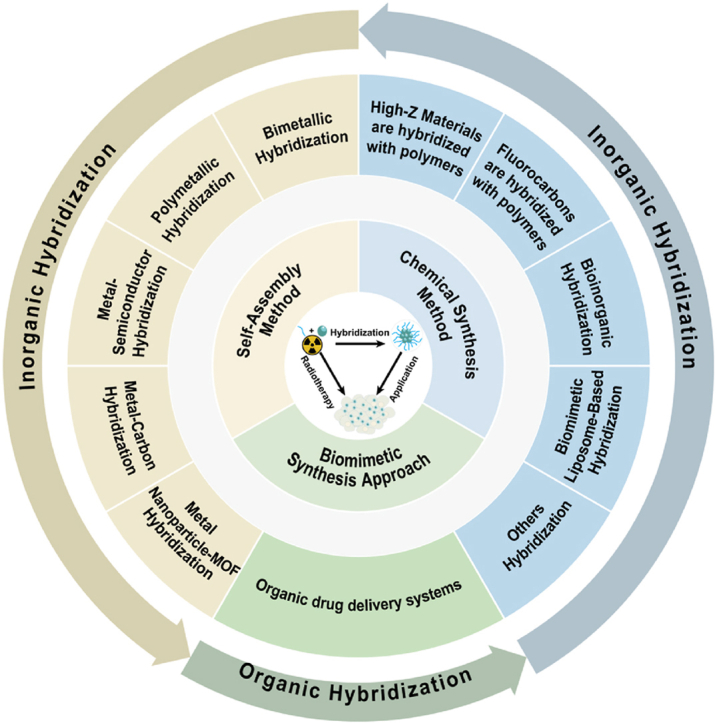

## 1 Introduction

Radiotherapy has long served as a cornerstone in cancer treatment, with over 40 % of cancer patients relying on it to inhibit tumor proliferation and achieve effective disease control [1,2]. However, conventional radiotherapy often encounters substantial challenges, including collateral damage to healthy tissues, radioresistance of tumor cells, and uncertainties in therapeutic outcomes [3]. Even so, radiotherapy remains one of the main modalities for cancer treatment. It utilizes high-energy ionizing radiation to induce DNA damage and initiate tumor cell death. Its therapeutic effectiveness mainly depends on the generation of ROS, which mediate double-strand breaks and oxidative stress in tumor cells. Nevertheless, the clinical efficacy of radiotherapy is frequently restricted by several limitations. These include tumor hypoxia, non-specific damage to adjacent healthy tissues, and the inherent radioresistance of some cancer types [4]. To tackle the above-mentioned issues, nanomaterial-based agents have come to the fore, resolving some of these problems. Common nanomaterial agents encompass gold nanoparticles, carbon-based nanomaterials, hafnium-based nanoparticles, gadolinium-based nanoparticles, bismuth-based nanoparticles, ruthenium-based nanoparticles, among others. Nevertheless, nanomaterial agents, particularly those based on single-component high atomic number (high-Z) metals, encounter challenges such as poor stability, low targeting efficiency, and inadequate ROS amplification [5,6].

Hybrid biomaterials-based radiosensitizers have emerged as a promising approach to address these challenges. By combining organic and inorganic components with complementary functions, these systems improve biocompatibility, facilitate precise tumor targeting, and enhance therapeutic responses through synergistic effects. The inclusion of catalytic elements, oxygen carriers, or responsive polymers enables the modulation of the tumor microenvironment, especially the alleviation of hypoxia and the promotion of ROS generation [[7], [8], [9], [10]]. These multifaceted benefits render hybrid systems particularly effective in surmounting the limitations of traditional methods. Consequently, they hold great promise for enhancing radiosensitization and improving clinical outcomes. In recent years, nanomedicine has come to the fore, offering promising strategies to boost the efficacy and precision of radiotherapy [11].

Due to their nanoscale size, nanomaterials enhance the enhanced permeability and retention (EPR) effect in tumor tissues, thereby promoting the accumulation and retention of therapeutic agents at the tumor site [[12], [13], [14], [15]]. This unique property confers a significant advantage for the development of radiosensitizers, as it enables efficient delivery of therapeutic agents to cancer cells [[16], [17], [18], [19], [20], [21]]. Moreover, nanomaterials exhibit additional advantages, such as a high surface area-to-volume ratio that facilitates enzyme-like reactions [22], customizable surface chemistries for improved biocompatibility [23], and the ability to mitigate radiotherapy resistance factors in the tumor microenvironment [24]. These attributes render them ideal candidates for enhancing radiotherapy efficacy and have been extensively investigated. Extensive research on nanoradiosensitizers has elucidated multiple radiosensitization mechanisms, including ROS generation [25], enhanced DNA damage [[26], [27], [28]], and modulation of the tumor microenvironment [[29], [30], [31]]. However, single-material nanoradiosensitizers, which often possess limited or singular radiosensitization mechanisms, continue to encounter challenges related to biocompatibility, stability, and targeting specificity.

For example, within a single material, gold nanoparticles (AuNPs) often face limitations in biological environments due to their instability, such as aggregation or degradation. Nevertheless, through hybridization techniques, AuNPs can form stable nanostructures, thereby improving their stability in biological settings. For instance, AuNPs can be combined with other nanomaterials, including lipids, sugars, and polymers, to form nanoemulsion or nanovesicle structures. These structures are not only stable but also capable of preventing aggregation, ensuring a longer circulation time. Moreover, they can regulate drug release or enable targeted therapy. Undoubtedly, this addresses the shortcoming of single materials, which typically possess only a single function, such as the generation of reactive oxygen species or radiation absorption. Hybrid materials enhance the therapeutic effect by integrating multiple functions, such as drug delivery and targeted therapy. For example, hybrid materials can enhance tumor targeting specificity by incorporating targeting ligands, such as folic acid [32,33] or peptides. Thus, through hybridization, these materials acquire enhanced functionalities, such as superior targeting ability, controlled-release mechanisms, and advanced imaging guidance, rendering them more effective in radiotherapy applications.

Despite the rapid expansion of research on hybrid nanomaterials, a comprehensive review of their radiosensitization effects remains scarce. Therefore, systematically summarizing the synthesis strategies of these hybrid nanomaterials and the advancements in their radiosensitization effects is of great significance.

**Scheme 1:**
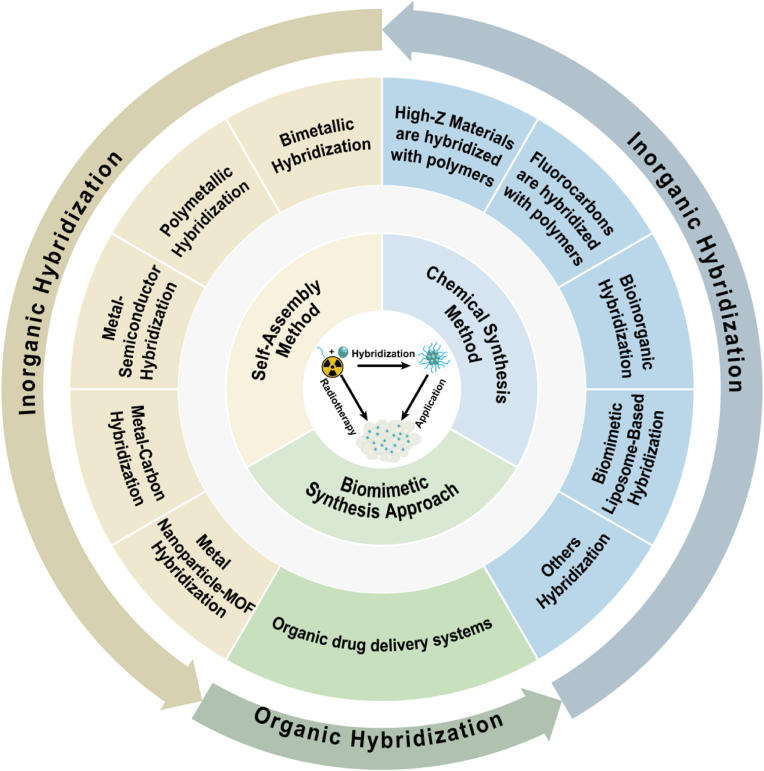
Overview of the classification, synthesis approaches of hybrid nanomaterials, and their applications in radiosensitization.

In this review, we first provide a comprehensive overview of hybrid biomaterials-based radiosensitizers and categorize the synthesis techniques for hybrid biomaterials-based radiosensitizers based on their distinct characteristics into three main types: self-assembly, chemical synthesis, and biomimetic synthesis. Subsequently, we classify the components of hybrid biomaterials-based radiosensitizers into inorganic and organic materials. Most hybrid materials take advantage of the unique benefits of inorganic materials. For instance, high-Z metals can enhance X-ray absorption and secondary electron generation, thus amplifying radiation-induced damage in tumor cells. At the same time, these hybrid materials also incorporate the advantages of organic materials, including excellent biocompatibility and multifunctionality. This combination enables the development of radiosensitizers that not only maximize therapeutic efficacy but also function through diverse mechanisms, such as enhancing ionizing radiation effects, alleviating tumor hypoxia, and depleting GSH. Finally, we explore the potential applications and future opportunities of these materials in the multidisciplinary biomedical field, providing valuable insights into the advancements of synthesis technologies for next-generation hybrid biomaterials-based radiosensitizers (Scheme 1).

## 2 Synthesis approaches for hybrid biomaterials-based radiosensitizers

Hybrid biomaterials-based radiosensitizers incorporate multiple sensitization mechanisms, thereby addressing the limitations of single-type sensitizers and enhancing the efficacy of radiation therapy. Through strategic synthesis approaches, hybrid biomaterials-based radiosensitizers markedly improve the radiation sensitization effects on tumors. To further optimize their performance, a variety of synthesis methods have been explored, each providing distinct advantages in terms of functional molecule integration, synthesis stability, and biological activity. This section presents a comprehensive review of the synthesis methods for hybrid biomaterials-based radiosensitizers, including self-assembly, chemical synthesis, and biological synthesis, with an emphasis on their unique contributions and potential to advance radiation therapy.

### 2.1 Self-assembly approach for hybrid biomaterials-based radiosensitizers

Self-assembly is a process wherein molecules spontaneously organize into well-defined structures via non-covalent interactions, such as hydrogen bonding, van der Waals forces, and electrostatic interactions [34]. This process is governed by mechanisms including hydrophobic effects, electrostatic interactions, π-π stacking, and templating effects [35]. Through these mechanisms, molecules can self-assemble into diverse structural forms, such as micelles [[36], [37], [38]], vesicles [39,40], and biologically mineralized nanoparticles [41,42]. Each of these structures provides distinct advantages for targeted and efficient delivery in radiation therapy, thereby significantly enhancing its efficacy.

**Fig. 1:**
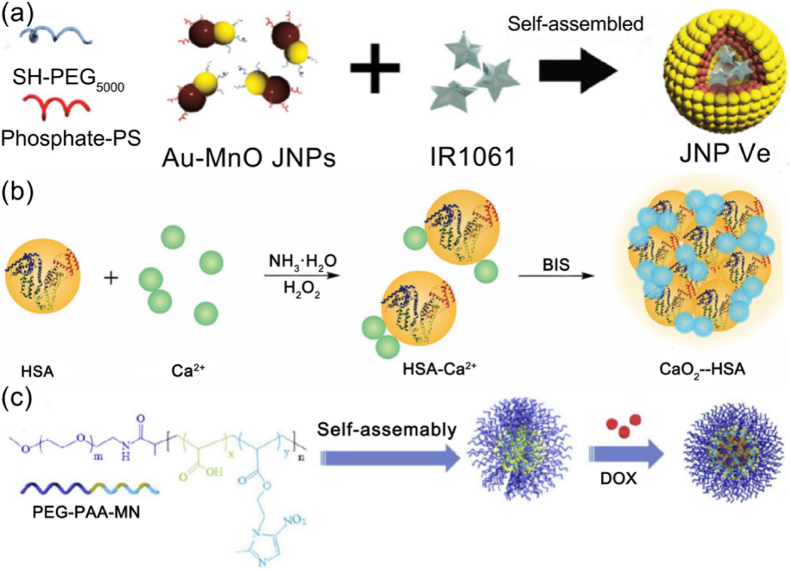
a) Schematic illustration of the fabrication of JNP Ve. Reproduced with permission [48]. Copyright 2021, Wiley-VCH GmbH. b) Schematic diagram of CaO_2_-HSA synthesis. Reproduced with permission [57]. Copyright 2024, Wiley-VCH GmbH. c) Schematic illustrations of pH/hypoxic dual-responsive DOX/PEG-PAA-MN micelles. Reproduced with permission [60]. Copyright 2020, Elsevier.

The self-assembly synthesis of hybrid biomaterials-based radiosensitizers typically involves techniques such as oil-in-water emulsification, biomineralization, and chemically induced self-assembly. These methods are employed to integrate gold [43,44], platinum [45], and other metal nanoparticles [46,47] —renowned for their unique surface plasmon resonance properties —with other functional nanomaterials. The resultant hybrid structures can markedly enhance the efficacy of radiotherapy. For instance, Lin et al. [48] utilized the oil-in-water emulsification method to self-assemble gold nanoparticles and manganese oxide into hybrid nanovesicles (JNP Ves) (Fig. 1a). Manganese oxide was deposited onto gold nanoparticles via heteroepitaxial growth, followed by dual-surface modification with hydrophilic thiolated polyethylene glycol (SH-PEG) and hydrophobic phosphate (P-PS) polymers. This dual modification enabled the nanoparticles to self-assemble into hybrid nanovesicles during the emulsification process. Upon exposure to the high GSH environment within tumor tissues, GSH reacts with specific chemical bonds in the nanovesicles, such as disulfide bonds, reducing them to thiol groups (-SH). This reaction triggers the disassembly or disruption of the nanovesicle structure. Subsequently, the hybrid nanovesicles decompose into smaller nanoparticles, releasing their active components. This disassembly facilitates deeper penetration into tumor tissues, thereby improving treatment precision and efficacy while minimizing collateral damage to surrounding healthy tissues. In summary, this oil-in-water self-assembly synthesis method exhibits significant radiosensitization effects, offering substantial advantages in radiotherapy.

This self-assembly approach and the resultant hybrid nanovesicles address several crucial limitations of conventional single-material nanoradiosensitizers. Traditional single-material systems, like gold nanoparticles, can enhance radiation absorption. However, they frequently encounter problems such as poor stability caused by aggregation in biological settings, which restricts their therapeutic potential. Additionally, these systems generally act through a single mechanism, such as radiation absorption or ROS generation, thereby limiting their overall effectiveness. By combining manganese oxide with gold nanoparticles, the hybrid system improves both stability and functionality [[49], [50], [51], [52], [53], [54]]. The manganese oxide component not only enhances the structural integrity of the nanoparticles but also provides additional radiosensitization through its own mechanisms, thereby creating a synergistic effect. Moreover, the dual-surface modification (SH-PEG and P-PS) of the nanoparticles enables them to maintain stability and dispersion, addressing the aggregation problem frequently encountered in traditional single-material systems. Additionally, the hybrid nanovesicles' ability to respond to the tumor microenvironment (such as disassembling upon exposure to GSH) allows for targeted and controlled drug delivery, facilitating deeper tissue penetration and improved specificity [55,56]. These characteristics render the hybrid system significantly more versatile and efficient in radiotherapy when compared to traditional single-material radiosensitizers. The latter are generally less adaptable and less effective in tumor targeting.

In conclusion, the water-in-oil-based self-assembly synthesis method enables these biological materials to achieve remarkable radiosensitizing effects. It overcomes the limitations of traditional single-material nano-radiosensitizers, enhances stability, functionality, and targeting specificity, thereby improving the therapeutic efficacy and minimizing damage to healthy tissues.

In addition, the biomineralization-induced self-assembly approach not only enhances the stability of hybrid biomaterials-based radiosensitizers but also significantly boosts ROS production and alleviates tumor hypoxia. For instance, in the synthesis of biomineralization-induced self-assembly hybrid biomaterials-based radiosensitizers, Gong et al. [57] utilized human serum albumin (HSA) as a biological template (Fig. 1b). By guiding the deposition of calcium peroxide (CaO_2_) on the HSA surface and spontaneously forming an ordered structure, stable coordination bonds were established between HSA and CaO_2_ nanoparticles, leading to the formation of a structurally robust CaO_2_-HSA nanocomposite.

Compared to traditional single-material radiosensitizers, which often suffer from issues such as poor stability, limited ROS production, and insufficient tumor hypoxia alleviation, hybrid biomaterials-based radiosensitizers offer significant advantages. Traditional single-material systems, such as gold nanoparticles, may efficiently absorb radiation but often lack the ability to generate sufficient ROS or improve the oxygenation of hypoxic tumor regions, which limits their therapeutic effectiveness. In contrast, the biomineralization-induced self-assembly approach integrates multiple therapeutic mechanisms into a single system. By incorporating calcium peroxide (CaO_2_) into the HSA template, the hybrid system not only enhances ROS production but also facilitates oxygen generation through the decomposition of CaO_2_, addressing the tumor hypoxia problem that traditional single-material systems cannot overcome. Furthermore, the stable coordination bonds between HSA and CaO_2_ nanoparticles significantly improve the stability of the nanocomposite, preventing aggregation and enhancing its long-term efficacy.

During radiotherapy, this biomineralization-induced self-assembly strategy effectively integrates calcium overload, oxygen generation from CaO_2_ decomposition, and elevated ROS levels, thereby amplifying the cytotoxic effects of X-rays on tumor cells [58,59]. This approach significantly increases the therapeutic efficacy compared to traditional single-material systems that rely solely on one or two mechanisms of action, such as radiation absorption or ROS generation. Moreover, the self-assembled CaO_2_-HSA nanocomposite exhibits improved biocompatibility, as the use of HSA ensures better interaction with the biological system, making it safer for use in vivo and reducing potential toxicity compared to synthetic traditional single-material nanoparticles. In summary, this biomineralization-induced self-assembly strategy not only improves the stability, functionality, and biocompatibility of the hybrid radiosensitizer but also enhancing the overall efficacy and reducing side effects during radiotherapy.

Furthermore, the core-shell micelle self-assembly method improves the responsiveness and targeting of hybrid nanosensitizers within the tumor microenvironment. As a result, this method provides substantial advantages for radiosensitization.

Compared with traditional single-material radiosensitizers, which usually lack the capacity to adapt to the dynamic tumor environment, hybrid systems provide enhanced functionality and responsiveness. Single-component systems, like gold nanoparticles or metal oxide nanoparticles, may exhibit excellent radiation absorption or ROS generation capabilities. However, they generally fail to achieve precise targeting and responsive reactions within tumor tissues. In contrast, the core-shell micelle self-assembly method incorporates dual-responsive properties, enabling enhanced adaptability within the tumor microenvironment. For example, Weng et al. [60] developed a spontaneously assembled stable core-shell structure composed of pH-sensitive polyethylene glycol-polyacrylic acid (PEG-PAA) block copolymers and hypoxia-sensitive metronidazole (MN). (Fig. 1c). This hybrid system leverages the pH sensitivity of PEG-PAA to respond to the acidic conditions in tumors and the MN to mimic oxygen effects. This is a critical limitation in traditional single-material radiosensitizers.

It is crucial to emphasize that although self-assembled structures operate efficiently under ideal physiological circumstances, such as the localized tumor microenvironments featuring acidic pH or heightened GSH levels, the non-covalent interactions (e.g., hydrogen bonds, van der Waals forces) responsible for driving self-assembly are intrinsically weak. This structural vulnerability may result in premature disassembly or diminished mechanical integrity when these structures are exposed to the intricate systemic environment of the human body, such as during blood circulation or in the presence of serum proteins. Consequently, while self-assembly improves functional responsiveness and biocompatibility, enhancing the in vivo structural stability remains a critical hurdle for their clinical translation.

### 2.2 Chemical synthesis approach for hybrid biomaterials-based radiosensitizers

The chemical synthesis method denotes the systematic process of converting various raw materials or precursors into the target product via controlled chemical reactions. During this process, reactants undergo specific chemical transformations, such as redox reactions, acid-base reactions, condensation reactions, and hydrolysis reactions, to generate new substances with defined composition, structure, and properties. Key techniques employed in this method include the co-precipitation methods, hydrothermal (solvothermal) synthesis, and sol-gel approach, which enable precise regulation of the size, shape, and chemical composition of the synthesized hybrid materials.

**Fig. 2:**
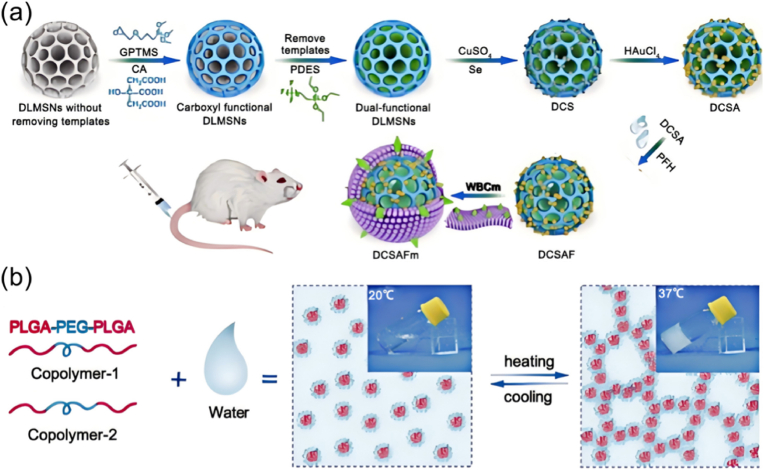
a) Schematic illustrations for the preparation and PTT-boosted RT synergistic effects of DCSAFm against breast cancer. Reproduced with permission [67]. Copyright 2022, Elsevier. b) Schematic illustrations depict the process where the blending copolymers self-assemble into micelles in water, followed by the formation of a percolated micelle network through micelle aggregation upon heating, resulting in the sol-gel transition. Reproduced with permission [74]. Copyright 2020, Elsevier.

The co-precipitation method represents one of the most classical and widely adopted techniques in the chemical synthesis of hybrid materials [[61], [62], [63]]. Radiosensitizers prepared via this method enable the simultaneous precipitation of multiple components within a solution, thereby simplifying reaction conditions while effectively achieving uniform dispersion and synergistic interactions among functional components, which ultimately enhances radiosensitization performance [[64], [65], [66]]. For instance, Guo et al. [67] developed hybrid nanoplatforms by in situ depositing Cu_2-x_Se and Au nanoparticles onto the surface of bifunctional dendritic large-pore mesoporous silica nanoparticles (DLMSNs), forming a Cu-Se-Au alloy (Fig. 2a). This co-precipitation approach leveraged the redox reaction between Cu^+^ and Au^3+^ to generate Cu^2+^ and Au^0^, ensuring stable nanoparticle loading, precise control over particle size, and uniform distribution across the nanoplatform. Consequently, this resulted in a robust and highly efficient nanotherapeutic system. This strategy integrates the radiosensitizing properties of high-Z metals with oxygen-carrying perfluorocarbon encapsulation, significantly amplifying tumor radiosensitivity.

Compared with traditional single-material systems, which frequently encounter instability and aggregation problems, the hybrid system guarantees greater structural integrity. Traditional single-material systems, like pure gold or copper nanoparticles, generally struggle to maintain stability in physiological conditions due to aggregation. This aggregation can result in a decrease in therapeutic efficacy. In this instance, the hybrid approach, which combines Cu_2-x_Se and Au nanoparticles with mesoporous silica, not only stabilizes the nanoparticles but also ensures a uniform size and distribution, thus overcoming the common instability issues observed in traditional single-material systems.

In contrast to the co-precipitation method, the hydrothermal (solvothermal) method exploits the high temperature and pressure to significantly improve the crystallinity [68,69], morphological control [70], and dispersibility of the synthesized material [71], thereby providing superior performance in the synthesis of hybrid radio-sensitive materials.

Traditional single-material systems frequently encounter problems such as poor crystallinity, irregular morphology, and aggregation, which diminish their efficacy. Conversely, the hydrothermal method enables precise control of nanoparticle growth, guaranteeing uniform size, high crystallinity, and enhanced dispersibility. This leads to more stable and efficient therapeutic effects. For example, Zang et al. [72] synthesized biocompatible polyvinylpyrrolidone (PVP)-modified hybrid Bi_2_WO_6_ nanosheets through a hydrothermal chemical synthesis method to enhance radiotherapy as a hybrid radiosensitizer. The process involved dissolving Bi(NO_3_)_3_·5H_2_O and Na_2_WO_4_·2H_2_O adjusting the pH, and subjecting the mixture to high temperature and pressure, which promoted uniform growth and stabilized the nanoparticles.

This approach guarantees high-quality, uniformly dispersed, and highly crystallized nanosheets. It effectively circumvents the aggregation and size fluctuations commonly encountered in traditional single-material systems. Under X-ray irradiation, the Bi_2_WO_6_ nanosheets with excellent crystal structure and dispersibility exhibited strong photocurrent response and radiocatalytic activity, effectively enhancing tumor radiotherapy by generating highly reactive oxygen species, such as hydroxyl radicals (•OH), through the high-Z metal.

While the hydrothermal (solvothermal) approach excels in controlling crystallinity, morphology, and dispersibility, the sol-gel approach provides superior control over the homogeneity and porosity of synthesized hybrid radio-sensitizers [73], providing a smoother transition for the development of efficient radiotherapy materials.

Traditional single-material systems, like metal nanoparticles, frequently encounter challenges in precisely controlling the porosity and uniformity of their structure. This limitation can impede their capacity to yield consistent therapeutic outcomes. Conversely, the sol-gel method enables meticulous control over the homogeneity and porosity of hybrid systems, thereby enhancing their stability and performance in radiotherapy. For example, Yang et al. [74] employed a sol-gel method to mix PLGA-PEG-PLGA block copolymers of varying compositions in water. At low temperatures, PLGA-PEG-PLGA copolymers spontaneously form extremely concentrated, homogeneous micelles in water. These micelles, consisting of a hydrophobic PLGA core and a hydrophilic PEG shell, maintain a sol-like state of solution at low temperatures. As the temperature increases, the enhanced hydrophobic interaction between the PLGA-PEG-PLGA micelles leads to the aggregation of additional micelles, forming a percolating micelle network (Fig. 2b). This network constrains molecular motion and facilitates the transition from sol to thermosensitive gel, while enabling better control of the gel porosity.

Traditional single-material radiosensitizers typically lack the flexibility to undergo such controlled transitions or adapt to the tumor microenvironment. In contrast, the sol-gel method offers a more dynamic system, allowing for controlled drug release and enhancing the overall functionality of the radiosensitizer. Subsequently, by loading fatty acid-modified gemcitabine derivatives, a gemcitabine (Gem) sustained-release system based on thermosensitive hydrogel was synthesized. The controlled porosity allows GemC16 to be released in a linear and continuous manner, exhibiting superior long-term drug release properties that significantly enhance GemC16’s efficacy in chemoradiotherapy.

This improved control over drug release and material properties gives the sol-gel method a clear advantage over single-component systems. As a result, it is an effective strategy for synthesizing high-performance hybrid biomaterials-based radiosensitizers.

Overall, chemical synthesis strategies, such as co-precipitation, hydrothermal (solvothermal), and sol-gel methods, provide strong control over the physicochemical properties of hybrid biomaterials-based radiosensitizers. These methods allow for precise regulation of particle size, crystallinity, morphology, and porosity, thereby enhancing dispersibility, drug loading capacity, and ROS-generating efficiency. Compared with traditional single-material radiosensitizers, hybrid materials synthesized through chemical methods exhibit improved tumor targeting, controlled drug release, and higher therapeutic efficacy.

Nonetheless, these techniques do have limitations. For example, hydrothermal and solvothermal methods typically demand high-pressure and high-temperature conditions. This can restrict their compatibility with temperature-sensitive biomolecules and make scale-up more complex. The sol-gel method, although beneficial for porosity control, may involve time-consuming gelation and aging processes that are hard to standardize. Moreover, the co-precipitation method, despite its simplicity, can be highly sensitive to pH, ionic strength, and reaction kinetics, leading to batch-to-batch variability or poor reproducibility. These limitations present challenges for industrial-scale production and clinical translation.

Notwithstanding these challenges, chemical synthesis continues to be a flexible and promising approach for the design of advanced hybrid biomaterials-based radiosensitizers with adjustable properties. This paves the way for future innovation in radiotherapy.

### 2.3 Biomimetic synthesis approach for hybrid biomaterials-based radiosensitizers

**Fig. 3:**
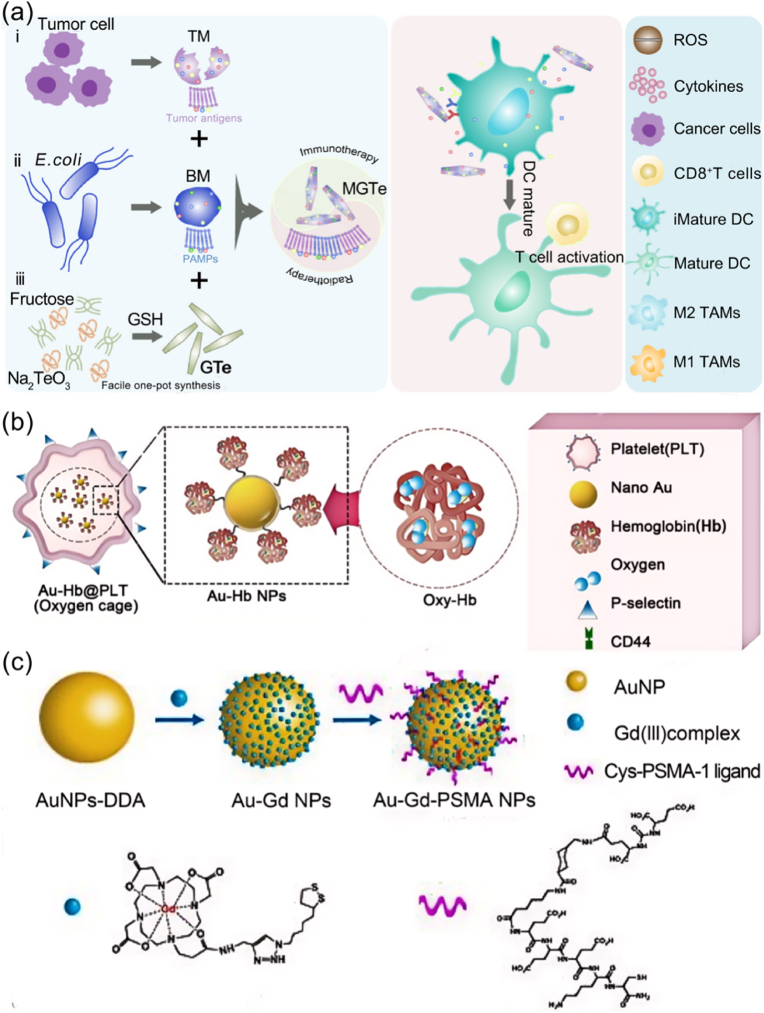
a) Preparation of MGTe. TM, BM, and GTe are mixed and repeatedly extruded to obtain MGTe. Reproduced with permission [80]. Copyright 2022, Elsevier. b) Schematic diagram of Au-Hb@PLT synthesis. Reproduced with permission [81]. Copyright 2020, American Chemical Society. c) Schematic diagram of Au-Gd-PSMA NPs synthesis. Reproduced with permission [82]. Copyright 2020, American Chemical Society.

The biomimetic synthesis approach for hybrid radiotherapy sensitizers pertains to a technique that utilizes biomimetic principles to synthesize hybrid materials possessing radiosensitizing attributes by emulating the natural reaction mechanisms or structural characteristics witnessed in biological systems. This methodology typically encompasses the employment of tumor cell membranes or other biological constituents, such as proteins [75,76], antigens [77], and antibodies [78,79], to simulate natural biological processes via techniques like biosynthesis and biomembrane coating. This approach enables the generation of hybrid biomaterials-based radiosensitizers with specific biomimetic traits and enhanced biocompatibility. For example, Pan et al. [80] disclosed the development of a biomimetic nanoplatform (Fig. 3a), MGTe, by fusing tumor membranes (TM) and bacterial outer membranes (BM) onto the surface of GSH-modified tellurium (Te) nanoparticles (GTe). Through sonication and repeated extrusion, TM and BM were integrated onto the GTe surface, forming MGTe nanoplatforms with a biomembrane coating.

When only a single material system, GTe, is employed for treatment, several limitations often arise. For instance, it typically exhibits poor biocompatibility and low targeting specificity. These drawbacks restrict its capacity to effectively interact with tumor cells and enhance the efficacy of radiotherapy. Conversely, the incorporation of biomembrane coatings in the MGTe system enhances both biocompatibility and targeting ability by capitalizing on the natural characteristics of tumor and bacterial membranes. This not only boosts the nanoparticles' ability to selectively accumulate in tumor tissues but also facilitates a more robust interaction with the tumor microenvironment.

This technique exploits the properties of biomembranes to augment the biocompatibility and targeting capacity of the material, thereby increasing the production of ROS and significantly enhancing the radiosensitization effect. Furthermore, the incorporation of biomembrane coatings also surmounts the functionality constraints of single-component systems. These systems typically depend on a single mechanism of action (such as radiation absorption or ROS generation). In this hybrid system, the biomembrane coating not only promotes ROS production but also heightens immune activation, offering a more comprehensive therapeutic outcome.

Therefore, this integrated approach overcomes the limitations of single-component radiosensitizers, including poor biocompatibility, low targeting capabilities, and inadequate functional performance. Thus, it boosts the radiosensitization effect and immune activation function, rendering it more versatile and effective in enhancing treatment outcomes.

In addition to improving biocompatibility by means of biomembrane coating, the functionalization of radiosensitizers with other biological materials also presents considerable advantages. This approach not only effectively enhances radiosensitivity but also reduces harm to normal tissues. This compensates for the limitations of the traditional single-material system of metal nanoparticles. The latter mainly concentrate on radiation absorption or reactive oxygen generation but cannot address problems such as tumor hypoxia or poor biocompatibility. Conversely, the hybrid method of functionalizing radiosensitizers with biological materials like hemoglobin and platelets overcomes these limitations. For instance, Xia et al. [81] fabricated gold nanoparticle-hemoglobin composite nanoparticles (Au-Hb@PLT) through a biofunctionalization strategy (Fig. 3b). This approach not only improves biocompatibility by utilizing platelets to prevent nanoparticles from being recognized by the immune system but also takes advantage of the oxygen-carrying ability of hemoglobin to relieve the hypoxic conditions commonly found in tumor microenvironments.

In an oxygen-rich milieu, thiol-modified hemoglobin (Hb) was initially combined with oxygen and then covalently bound to gold nanoparticles (AuNPs) to form Au-Hb composite nanoparticles, which were subsequently loaded onto platelets. This biofunctionalization approach overcomes the substantial limitation of traditional single-material systems. These traditional systems face difficulties in alleviating hypoxia and are typically less effective in oxygen-deprived tumor areas. The addition of hemoglobin significantly boosts the therapeutic effectiveness by increasing the oxygen availability within tumors, which is a crucial factor in radiosensitization.

This biofunctionalization method exploits the outstanding oxygen-carrying capacity of hemoglobin to alleviate hypoxia in the tumor microenvironment, while platelets enhance biocompatibility within biological tissues. Moreover, by ameliorating the hypoxic environment of the tumor, it can further enhance the radiosensitizing effect of high-Z metals in the composite material, thereby significantly increasing the sensitivity of tumor cells to X-rays. Consequently, the hybrid Au-Hb@PLT nanoplatforms overcome the key challenges of traditional single-material radiosensitizers by improving both their biocompatibility and radiosensitization potential. This makes them more effective in tumor treatment, especially in hypoxic regions that are often refractory to conventional therapies.

Although the hybrid Au-Hb@PLT platform exhibits excellent biocompatibility and effectively mitigates hypoxia, antibody-based bioconjugation approaches provide superior targeting capabilities. Single-material radiosensitizers, although effective in enhancing radiation absorption and ROS generation, frequently encounter limitations in targeting specificity. This often results in suboptimal therapeutic outcomes and potential harm to healthy tissues. Conversely, hybrid systems such as the Au-Gd-PSMA nanoparticles combine high-Z metals for radiosensitization with specific targeting ligands, thereby significantly enhancing targeting specificity. For instance, Luo et al. [82] developed a novel nanomaterial, namely Au-Gd-PSMA nanoparticles, designed for MRI-guided targeted radiotherapy of prostate cancer (Fig. 3c). These nanoparticles were synthesized by conjugating Gd(III) complexes and a prostate-specific membrane antigen (PSMA) targeting ligand (Cys-PSMA-1) onto AuNPs.

Initially, the surface of AuNPs was modified with a 1, 2-dithiolane anchor, which firmly binds the Gd(III) complexes and enhances colloidal stability, maintaining the small size of the nanoparticles without the requirement for additional stabilizers. Traditional single-material systems frequently encounter challenges regarding colloidal stability. To maintain particle size and prevent aggregation, additional stabilizers are required. In this hybrid system, the utilization of the 1, 2-dithiolane anchor not only improves the stability of the nanoparticles but also allows for better control of their size, guaranteeing consistent performance during treatment.

This biomimetic synthesis method enables the Au-Gd-PSMA nanoparticles to effectively target prostate cancer cells, minimizing damage to the surrounding healthy tissues while maximizing therapeutic effects on cancer cells. By integrating the PSMA targeting ligand, this hybrid system provides enhanced tumor targeting capabilities compared to traditional single-material radiosensitizers. The latter usually lack such a high level of specificity and flexibility.

In conclusion, the biomimetic synthesis method provides a promising platform for the development of hybrid biomaterials-based radiosensitizers, which feature enhanced biocompatibility, targeting specificity, and multifunctionality. By means of strategies such as biomembrane coatings, biofunctionalization, and antibody conjugation, these systems imitate natural biological structures and functions to overcome the limitations of traditional single-material radiosensitizers. These limitations include poor targeting ability, low stability, and restricted therapeutic scope. These advancements allow for more precise drug delivery, effective tumor accumulation, and improved radiosensitization through multiple mechanisms, such as oxygen modulation, immune activation, and ROS generation.

At present, biomimetic radiosensitizers are in a developmental phase. They demonstrate outstanding performance in preclinical models, especially in dealing with hypoxia, promoting immune activation, and facilitating tumor-specific accumulation. Nevertheless, there are still challenges that need to be overcome for clinical translation. These challenges involve the potential immunogenicity of biological components (such as tumor cell membranes and platelets), the limited long-term stability under physiological conditions, and the variability of raw materials. The differences in cell membrane components and donor-derived platelet characteristics may affect batch consistency and functional reproducibility. Moreover, the multi-step fabrication process and strict quality control requirements impede scalability and reproducibility.

Future research ought to concentrate on enhancing the standardization of biomimetic material sources, optimizing synthesis procedures, and performing comprehensive in vivo safety evaluations. Resolving these issues will be of great significance for the wider clinical application of biomimetic hybrid biomaterials-based radiosensitizers and their incorporation into next-generation radiotherapy strategies.

To sum up, the three preparation methods, namely self-assembly, chemical synthesis, and biosynthesis, each present unique advantages and challenges. Table 1 compares these methods, emphasizing their respective strengths and limitations.

**Table 1:** Advantages and disadvantages of various preparation strategies for hybrid nanomaterials.

Preparation Strategies	Advantages	Disadvantages
Self-Assembly Approach	Structurally manipulable, highly targeted, and responsive to the tumor microenvironment, it can be precisely released in tumor tissues.	Highly sensitive to the environment and featuring poor stability, it demands high precision in process parameters.
Chemical Synthesis Approach	It features high controllability, a uniform structure, stable performance, and is suitable for large-scale production.	The reaction conditions are stringent, which might introduce toxic residues. The biocompatibility requires optimization.
Biomimetic Synthesis Approach	It features high biocompatibility, excellent targeting ability, ease of mimicking natural systems, and is suitable for enhancing bioavailability.	The synthesis process is intricate, and large-scale production poses challenges. Additionally, immunogenicity problems may arise.

## 3 Hybrid biomaterials-based radiosensitizer for enhanced radiotherapy

### 3.1 Inorganic hybrid radiosensitizer

Inorganic materials, especially high atomic number metals like gold and bismuth, augment radiation absorption, enhance energy deposition, and alleviate tumor hypoxia by virtue of their catalytic and enzyme-like characteristics, thereby rendering them efficient radiation sensitizers. Nevertheless, the application of single metal materials in radiotherapy still encounters limitations, such as a narrow mechanism of action and restricted therapeutic efficacy. To surmount these deficiencies, inorganic hybrid radiation sensitizers have emerged [83]. By capitalizing on the synergistic effects of multiple physical properties, hybrid materials not only ameliorate the tumor microenvironment and boost radiation absorption but also facilitate more precise targeted therapy. Consequently, inorganic hybrid radiation sensitizers exhibit distinct superiority over traditional single-component sensitizers in these respects. This section classifies inorganic hybrid materials into five types: bimetallic, polymetallic, metal-semiconductor, metal-carbon, and metal nanoparticle-MOF hybrids. The mechanisms and advantages of these types are deliberated, providing valuable perspectives for optimizing material applications and guiding future innovations.

#### 3.1.1 Bimetallic hybrid radiosensitizer for enhanced radiotherapy optimization

Bimetallic hybrid biomaterials-based radiosensitizers denote agents that augment the efficacy of radiotherapy through exploiting the synergistic impacts of diverse metals. Specifically, one metal, typically a high-Z metal like gold or platinum, is utilized to escalate the radiation energy deposition within tumors, resulting in the generation of more secondary electrons that directly or indirectly inflict damage on the DNA of tumor cells [84]. Another metal, such as platinum, iron, or ruthenium, might display catalase-like activity, catalyzing the decomposition of hydrogen peroxide in the tumor microenvironment into water and oxygen, thereby mitigating tumor hypoxia and further enhancing the radiosensitivity of tumor cells [85,86]. Consequently, bimetallic hybrid nanosensitizers that integrate these two mechanisms have been extensively investigated.

**Fig. 4:**
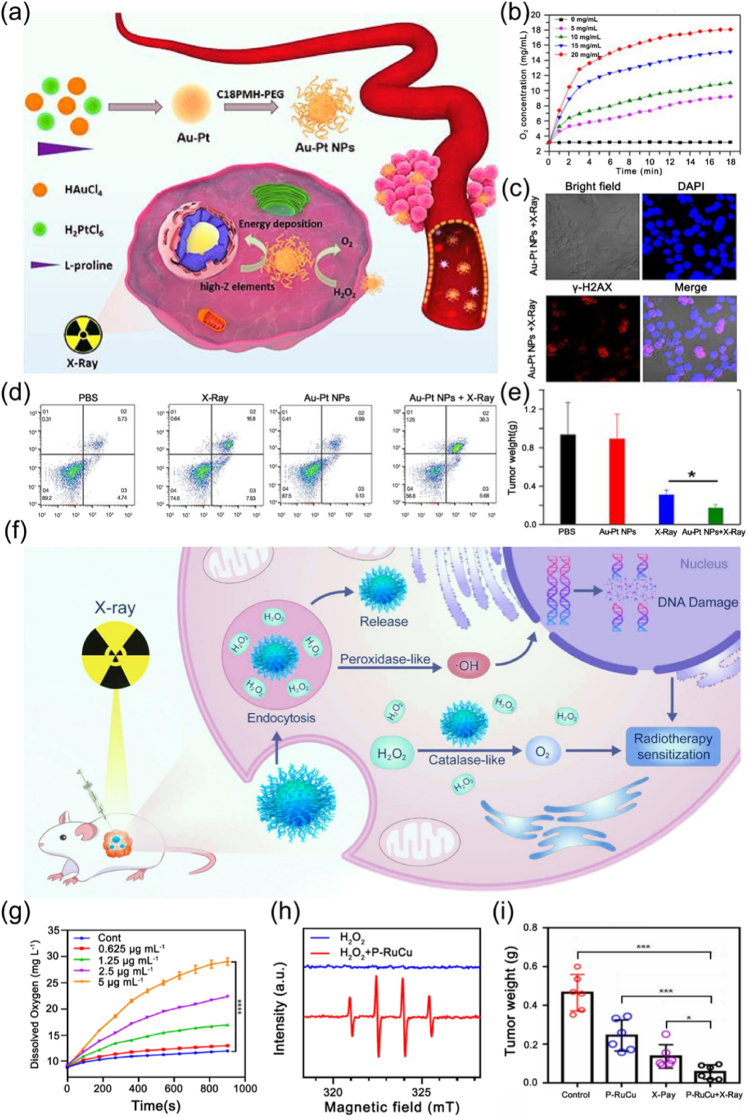
a) Schematic process and characterization of Au-Pt nanoparticles. Reproduced with permission [87]. Copyright 2021, Dove Medical Press. b) Oxygen production by Au-Pt nanoparticles with different concentrations in H_2_O_2_ solution. Reproduced with permission [87]. Copyright 2021, Dove Medical Press. c) Immunofluorescence image of phosphorylated H2AX (γH2AX) in 4T1 cells after DNA damage. Reproduced with permission [87]. Copyright 2021, Dove Medical Press. d) Flow cytometry analysis of apoptosis in 4T1 cells using Annexin V-FITC and PI staining after different treatments. Reproduced with permission [87]. Copyright 2021, Dove Medical Press. e) Tumor volumes of mice treated with various formulations. Reproduced with permission [87]. Copyright 2021, Dove Medical Press. f) Mechanism of P-RuCu as a therapeutic nanoenzyme to enhance cancer radiotherapy. Reproduced with permission [88]. Copyright 2022, Elsevier. g) O_2_ production in H_2_O_2_ solution with varying concentrations of P-RuCu at pH 6.5. Reproduced with permission [88]. Copyright 2022, Elsevier. h) ESR spectra of DMPO/·OH adducts in different reaction systems. Reproduced with permission [88]. Copyright 2022, Elsevier. i) Tumor weights of mice in each treatment group on day 12. Reproduced with permission [88]. Copyright 2022, Elsevier.

For instance, Yang et al. [87] fabricated Au-Pt bimetallic nanospheres that augment tumor DNA damage and mitigate tumor hypoxia (Fig. 4a). On the one hand, gold nanoparticles, being high-Z metals, display a strong nuclear attraction and a high inner electron density, prominently enhancing the probability of the photoelectric effect. In the photoelectric effect, X-ray photons transfer their energy completely to the inner electrons of gold atoms, and the deposited energy leads to the ejection of these electrons from the atom, generating high-energy electrons (such as secondary electrons). These electrons can directly inflict damage on the DNA of tumor cells or indirectly produce ROS, which also impairs tumor cell DNA. Concurrently, Au nanoparticles, as high-Z metals, enhance energy deposition and augment the lethality of tumor cells. On the other hand, platinum, as a noble metal, demonstrates high surface activity and outstanding catalytic properties, particularly at the nanoscale, where it offers numerous active sites that facilitate the dissociation and decomposition of H_2_O_2_ molecules on its surface. The resultant oxygen alleviates tumor hypoxia. Hence, in cellular experiments, these gold-platinum nanoparticles manifested potent oxygen-generating capabilities (Fig. 4b) and DNA damage effects (Fig. 4c and d). Analogously, in animal experiments, they exhibited exceptional tumor-reducing effects (Fig. 4e).

CAT-like metals primarily enhance radiotherapy by demonstrating catalase activity. Based on this, recent studies have discovered that certain metals not only maintain CAT-like properties but also exhibit POD-like characteristics, further augmenting their efficacy in radiotherapy. For instance, Hu et al. [88]synthesized a bimetallic RuCu hybrid radiosensitizer (P-RuCu) by integrating the high-Z metal Ru with the dual enzyme-like active metal Cu (Fig. 4f). The high-Z properties of Ru enhance radiotherapy by maximizing energy deposition in tumor cells through its interaction with X-rays. Simultaneously, Cu displays CAT and POD enzyme-like activities, allowing it to react with the overexpressed H_2_O_2_ in tumor cells to generate O_2_ (Fig. 4g), thereby alleviating tumor hypoxia. Additionally, Cu catalyzes the conversion of H_2_O_2_ into highly toxic ·OH molecules through chemical kinetics (Fig. 4h), enhancing ROS-mediated cytotoxicity. Therefore, the RuCu hybrid radiosensitizer (P-RuCu) also manifested significant therapeutic efficacy in a breast cancer animal model (Fig. 4i).

#### 3.1.2 Polymetallic hybrid radiosensitizer for enhanced radiotherapy optimization

Polymetallic hybrid biomaterials-based radiosensitizers are composite substances that integrate multiple metal elements to augment the efficacy of radiotherapy by virtue of the synergistic effects of their chemical and physical attributes, such as high atomic numbers, outstanding catalytic activity, and diverse electronic structures. Furthermore, the incorporation of metal elements with distinct lattice parameters, chemical properties, and electronic structures in polymetallic hybrid biomaterials-based radiosensitizers heightens the probability of forming heterostructures [89]. These heterostructures can further facilitate the generation of ROS [[90], [91], [92], [93]], thereby escalating the oxidative stress on tumor cells and enhancing the overall therapeutic effect of radiotherapy.

**Fig. 5:**
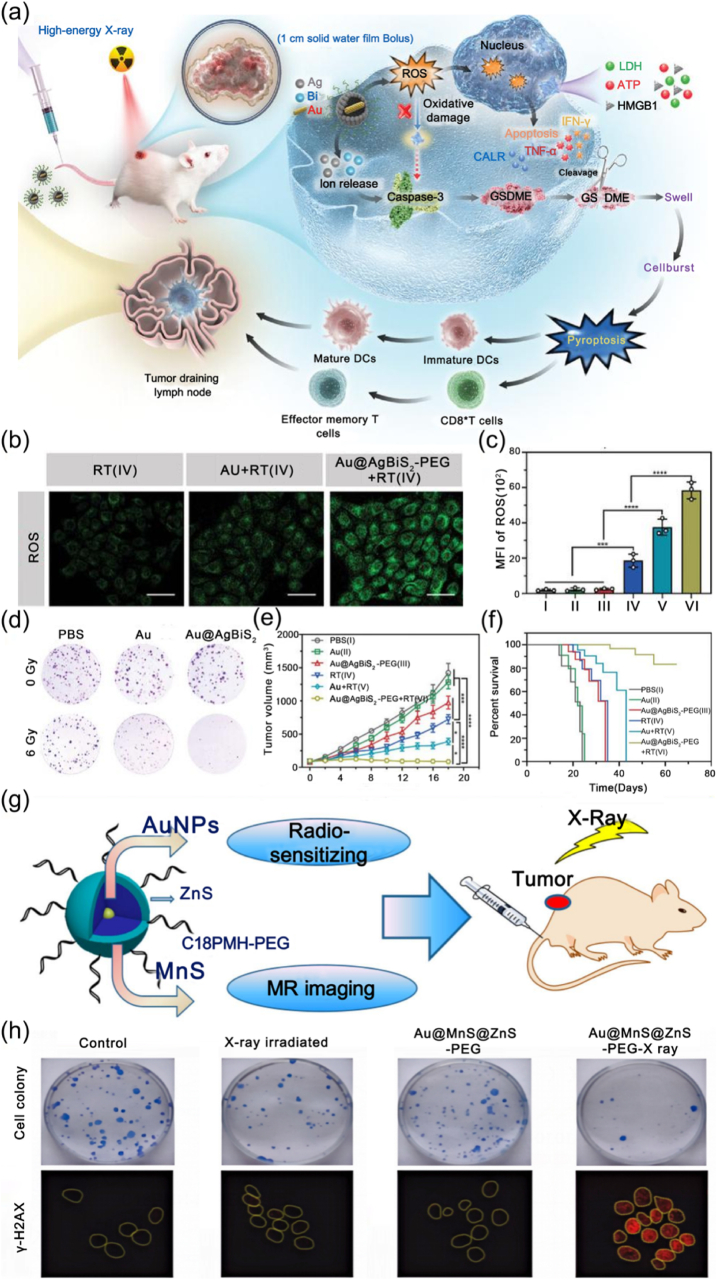
a) Diagram illustrating the antitumor application of Au@AgBiS_2_-PEG. Reproduced with permission [94]. Copyright 2021, Wiley-VCH GmbH. b) Confocal laser scanning microscopy was employed to observe ROS generation in 4T1 cells with different pretreatments. Reproduced with permission [94]. Copyright 2021, Wiley-VCH GmbH. c) Quantification of ROS levels in tumor sections. Reproduced with permission [94]. Copyright 2021, Wiley-VCH GmbH. d) Survival fraction of 4T1 cells after pretreatment with PBS, Au, or Au@AgBiS_2_-PEG under X-ray irradiation at 0 Gy and 6 Gy. Reproduced with permission [94]. Copyright 2021, Wiley-VCH GmbH. e) Tumor volumes of mice treated with various formulations. Reproduced with permission [94]. Copyright 2021, Wiley-VCH GmbH. f) Survival analysis of mice treated with different formulations. Reproduced with permission [94]. Copyright 2021, Wiley-VCH GmbH. g) Schematic representation depicting the design of Au@MnS@ZnS nanoparticles. Reproduced with permission [95]. Copyright 2016, American Chemical Society. h) Growth of 4T1 cancer cell colonies following 7 days after 4 Gy of X-ray irradiation. Immunofluorescent imaging of γ-H2AX foci in 4T1 cells incubated with or without Au@MnS@ZnS-PEG under X-ray irradiation. Reproduced with permission [95]. Copyright 2016, American Chemical Society.

For instance, Xiao et al. [94] fabricated a polymetallic hybrid nanoparticle, Au@AgBiS_2_, characterized by a heterostructure (Fig. 5a). This multimetallic hybrid nanosensitizer not only boosts radiotherapy via the deposition of ionizing radiation energy by high-Z metals like Au and Bi but also forms a Schottky barrier heterostructure through the hybridization of Au with the semiconductor AgBiS_2_, further facilitating radiosensitization. This Schottky barrier effectively promotes the separation of electrons and holes, minimizing their recombination. Consequently, more free electrons and holes are generated, which engage in ROS production, thereby elevating ROS levels (Fig. 5b and c).

Additionally, the Schottky barrier at the metal-semiconductor interface generates a potent local electric field, which further augments energy deposition, rendering X-rays or other radiation sources more efficient in delivering energy to tumor cells and resulting in enhanced DNA damage within tumor cells. Consequently, in cellular experiments, Au@AgBiS_2_ significantly elevates RT-mediated DNA damage via a ROS burst, thereby increasing cancer radiosensitivity and manifesting superior radiotherapy efficacy in comparison to single-material AuNPs (Fig. 5d). Similarly, in animal experiments, the hybrid material Au@AgBiS_2_ exhibited superior performance in reducing tumor size (Fig. 5e) and enhancing mouse survival rates (Fig. 5f) when compared with the single-material AuNPs.

In addition to providing superior radiosensitization *via* its heterostructure, polymetallic hybrid materials frequently incorporate metal elements possessing high magnetic or paramagnetic properties. These elements can markedly enhance the contrast of MR imaging, enabling tumors to be more clearly discernible in MR images, which facilitates the precise localization of tumors and enables more accurate in vivo imaging-guided radiotherapy. For instance, Li et al. [95] synthesized a polymetallic hybrid radiosensitizer, Au@MnS@ZnS, designed for MR imaging-guided precision radiotherapy (Fig. 5g). In this hybrid sensitizer, the Au core enhances RT by absorbing ionizing radiation, while the core/shell/shell structure of Au@MnS@ZnS forms a heterostructure that boosts the production of ROS, thereby increasing the lethality of tumor cells. Additionally, the existence of paramagnetic Mn^2+^ ions within the MnS structure enhances MR *T*_1_ contrast, making the tumor more clearly visible in MR images and guiding precise radiotherapy. Therefore, the Au@MnS@ZnS nanoparticles, through hybridization into a distinctive core-shell structure, achieved enhanced radiotherapy and MRI-guided dual functionality that is superior to single-component materials. This multifunctionality also leads to improved tumor cell-killing effects in cellular experiments. (Fig. 5h).

#### 3.1.3 Metal-semiconductor hybrid radiosensitizer for enhanced radiotherapy optimization

Metal-semiconductor hybrid biomaterials-based radiosensitizers are hybrid materials formed by combining metal nanoparticles with semiconductor materials, designed to synergistically enhance the effectiveness of radiotherapy. First, the photogenerated charge carrier properties of semiconductors are highly advantageous for radiosensitization [96]. Semiconductors can easily excite electrons from the valence band to the conduction band under electromagnetic radiation, leaving behind a vacancy in the valence band known as a “hole” [[97], [98], [99]]. These free electrons and holes can migrate and participate in reactions with water molecules or oxygen, generating ROS [100]. These ROS increase oxidative stress and DNA damage in tumor cells, ultimately enhancing the overall efficacy of radiotherapy. In addition, the high atomic number of metal nanoparticles increases X-ray absorption efficiency, concentrating more energy at the tumor site and further improving therapeutic outcomes. Thus, hybridizing metal nanoparticles with semiconductors maximizes their synergistic effects, significantly enhancing radiosensitivity.

**Fig. 6:**
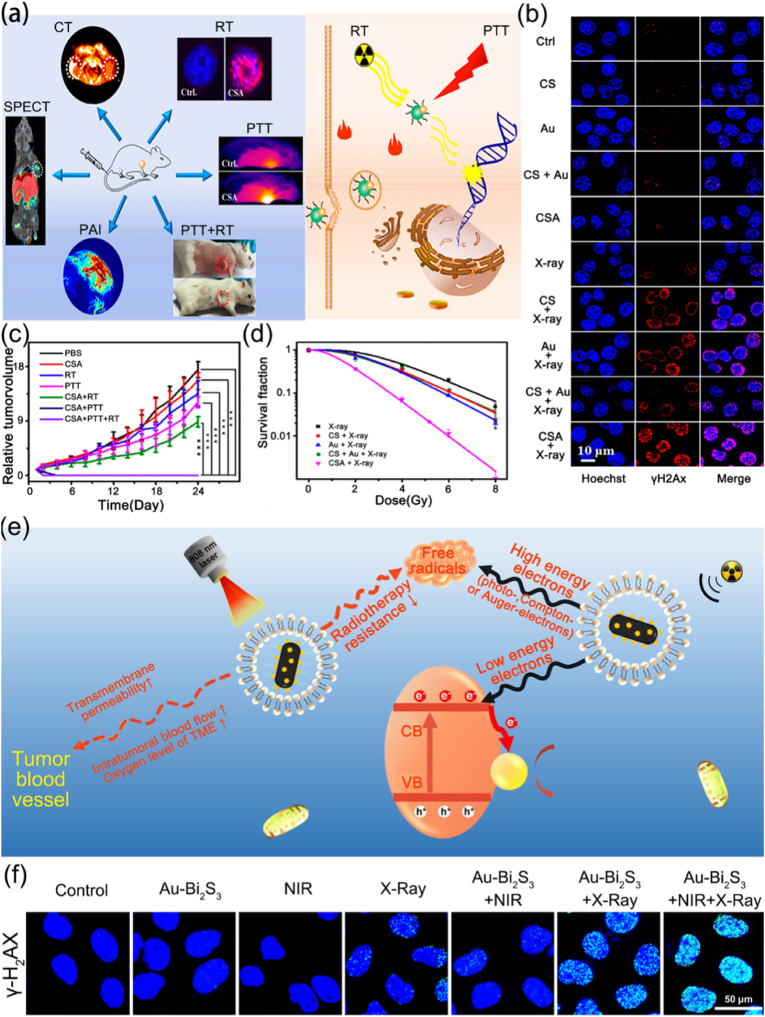
a) Application of heterogeneous CSA nanoparticles in PA/SPECT/CT multimodal imaging and synergistic radioactive photothermal therapy for cancer. Reproduced with permission [101]. Copyright 2019, American Chemical Society. b) DNA damage in 4T1 cells irradiated with 6 Gy X-rays in the presence or absence of CS, Au, CS + Au, and CSA nanoparticles. Reproduced with permission [101]. Copyright 2019, American Chemical Society. c) Changes in the survival of different groups of mice treated under different conditions for relative tumor volume. Reproduced with permission [101]. Copyright 2019, American Chemical Society. d) Survival of 4T1 cells treated with/without CS, Au, CS + Au, and CSA nanoparticles at different doses of X-rays. Reproduced with permission [101]. Copyright 2019, American Chemical Society. e) Schematic illustration of Therapeutic Process Based on Au–Bi_2_S_3_ HNSCs. Reproduced with permission [107]. Copyright 2019, American Chemical Society. f) DNA damage induced by different treatments in Au-Bi_2_S_3_ HNSCs. Reproduced with permission [107]. Copyright 2019, American Chemical Society.

For instance, Huang and his colleagues [101] synthesized copper selenide-gold nanocrystals (CSA) as a metal-semiconductor hybrid material featuring a heterostructure (Fig. 6a). The establishment of a heterostructure between metal nanoparticles and semiconductors boosts the generation and behavior of electron-hole pairs within the semiconductor under electromagnetic radiation. When exposed to electromagnetic radiation, electron-hole pairs are produced within the semiconductor material. The hybrid heterostructure, by offering multiple interfaces, effectively separates and captures these electrons and holes, lowering their recombination probability. This separation effect enhances the efficiency of ROS generation, thereby enhancing the radiosensitization effect. Furthermore, the high atomic number of gold nanoparticles considerably increases X-ray absorption, concentrating radiation energy at the tumor site. The Cu_2-x_Se-Au heterostructures work synergistically to enhance RT and PTT. Au has a high atomic number, which can boost radiation absorption and energy deposition at the tumor site during RT. Cu_2-x_Se has photothermal properties, which can convert near-infrared light into heat to promote additional tumor cell destruction during PTT. By combining these two properties, this heterostructure maximizes tumor damage while minimizing harm to surrounding healthy tissues, thus significantly improving the therapeutic efficacy. In cell experiments, the synergistic effect of the CSA hybrid material during radiotherapy demonstrated stronger sensitization than gold nanoparticles alone (Fig. 6b), leading to significant tumor cell destruction (Fig. 6c). In animal experiments, the hybrid radiosensitizer CSA significantly enhanced the survival rate of mice (Fig. 6d), indicating greater radiotherapy effectiveness compared to single-component radiosensitizers.

Heterojunctions have been demonstrated to provide superior radiosensitization in contrast to traditional heterostructures [93,[102], [103], [104]], owing to their more efficient charge separation and enhanced electron transfer, which result in increased generation of ROS and enhanced radiotherapy efficacy [90,93]. For instance, this phenomenon was manifested in an experiment conducted by Nosrati et al.where they synthesized a heterojunction Janus nanoparticle Bi_2_S_3_@BSA-Fe_3_O_4_-FA [105]. Heterojunctions form a potential difference or band alignment at the interface between two distinct semiconductor materials, facilitating the enhancement of charge separation efficiency. Combined with the photogenerated charge carrier properties of the semiconductor, this significantly boosts ROS generation. Additionally, the deposition of radiation energy by the high-Z metal further enhances radiosensitization. Moreover, the hybrid material utilizes Fe_3_O_4_ to generate ROS through a Fenton reaction via chemodynamic therapy, synergistically augmenting the radiotherapy-induced destruction of tumor cells. Animal experiments indicated that the heterojunction hybrid nanomaterial Bi_2_S_3_@BSA-Fe_3_O_4_-FA significantly enhanced the radiosensitivity of the treatment.

In contrast, Schottky-type heterostructured metal-semiconductor radiosensitizers exhibit superior charge separation efficiency and more efficient electron transfer, further augmenting radiosensitization [106]. For instance, Wang et al. [107] fabricated a Schottky-type heterostructured Au-Bi_2_S_3_ hybrid radiosensitizer (Au-Bi_2_S_3_ HNSC) through the i*n situ* growth of gold nanocrystals (Au NCs) on the surface of Bi_2_S_3_ nanorods (Fig. 6e). In the Schottky-type heterostructure, a built-in electric field is established at the Au-Bi_2_S_3_ metal-semiconductor interface. This intrinsic electric field originates from the disparity in Fermi levels between the metal and the semiconductor. This disparity results in electron transfer and band bending at the interface. This built-in electric field propels electrons and holes in opposite directions within the semiconductor, effectively minimizing electron-hole recombination, and thereby enhancing the generation of free electrons and ROS. This indicates that Schottky-type heterostructures are more efficacious in promoting cancer cell damage during radiotherapy. Additionally, the Schottky barrier formed at the interface further boosts the X-ray absorption of the high-Z metal Au nanoparticles, concentrating radiation energy more effectively at the tumor site and enhancing the radiotherapy-induced destruction of cancer cells. However, when compared with conventional semiconductor–semiconductor heterojunctions, Schottky-type heterostructures are more intricate to fabricate and provide less flexibility in adjusting key parameters such as band alignment, particle size, and interfacial properties. Consequently, in cell experiments, this Schottky-type heterostructured Au-Bi_2_S_3_ hybrid radiosensitizer manifested significant DNA damage in tumor cells (Fig. 6f), suggesting a strong radiosensitization capacity.

In conclusion, both heterojunction and Schottky-type heterostructures enhance radiosensitization by promoting charge separation and ROS production. Heterojunctions, formed between two semiconductors, provide greater flexibility in band alignment tuning and are suitable for integration into multifunctional platforms, such as those integrating photothermal or photodynamic therapy. They are especially beneficial in surface-modified or light-activated treatment strategies that require flexibility in material design.

Conversely, Schottky-type heterostructures, formed at the metal–semiconductor interface, generate an internal electric field that effectively inhibits electron-hole recombination, resulting in stronger ROS generation under X-ray irradiation. This makes them more effective in deep-tissue radiotherapy scenarios, where optical activation is limited and reliance on X-rays is dominant. However, their limited tunability and more stringent interface requirements may restrict their adaptability across different combination therapies. Therefore, the choice between these two structures should take into account specific clinical requirements, such as tissue depth, available energy sources (light versus X-ray), and the need for multimodal treatment.

#### 3.1.4 Metal-carbon hybrid radiosensitizer for enhanced radiotherapy optimization

Metal-carbon hybrid biomaterials-based radiosensitizers constitute a category of composite materials formed through the combination of metal nanomaterials with carbon-based structures (such as carbon quantum dots or graphdiyne) for the purpose of synergistically enhancing the efficacy of radiotherapy. These sensitizers not only exploit the properties of high atomic number (Z) or enzyme-like activity of metals to enhance radiosensitization but also draw benefits from hybridization with carbon nanomaterials, which endows them with an abundance of functional groups, chemical stability, and diverse modification possibilities [[108], [109], [110], [111]]. This facilitates the overcoming of the surface inertness and limited modifiability of metals, leading to more stable materials that further augment the effectiveness of radiotherapy [112].

**Fig. 7:**
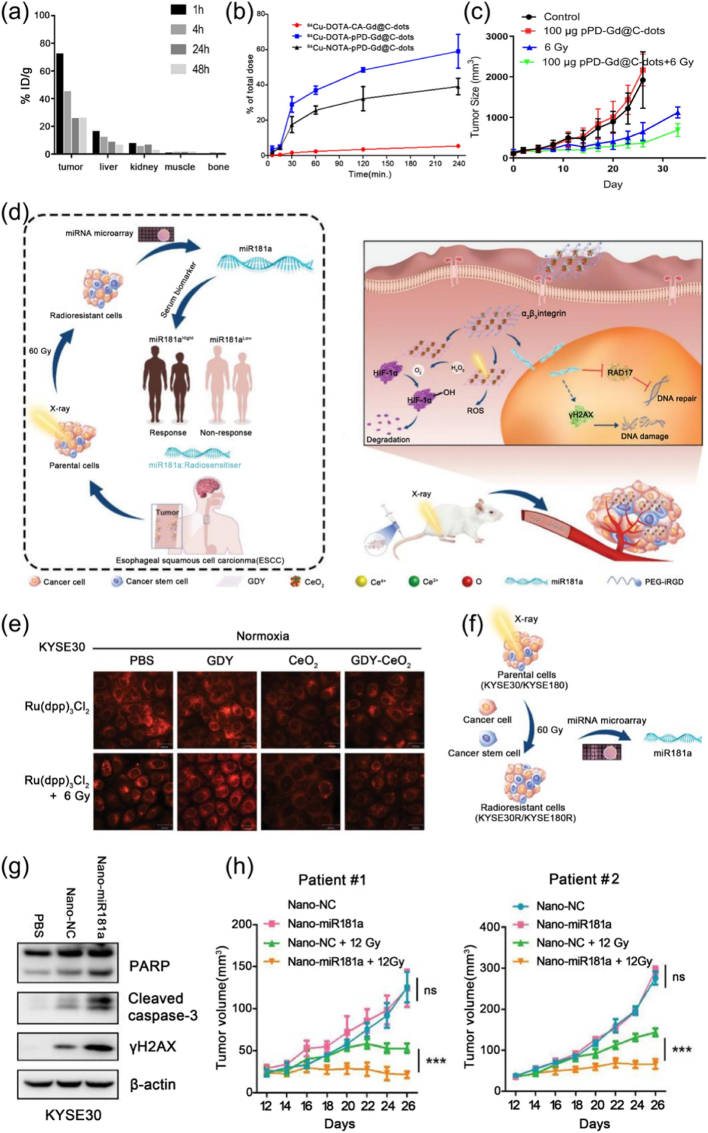
a) Comparison of absorption in various organs at different times after intratumoral injection of ^64^Cu-DOTA-pPD-Gd@C-dots. Reproduced with permission [113]. Copyright 2021, Springer Nature. b) Cellular uptake analysis of ^64^Cu-labeled Gd@C-dots in H1299 cells. Reproduced with permission [113]. Copyright 2021, Springer Nature. c) Tumor growth curve of the H1299 tumor model after X-ray radiotherapy. Reproduced with permission [113]. Copyright 2021, Springer Nature. d) Illustration of the multiple radiosensitizing mechanisms of GDY-CeO_2_ loaded miR181a on ESCC cells. Reproduced with permission [114]. Copyright 2021, Wiley-VCH GmbH. e) Confocal microscopy was used to detect the intracellular O_2_ level in KYSE30 cells treated with different treatments. Reproduced with permission [114]. Copyright 2021, Wiley-VCH GmbH. f) Schematic representation of miR181a expression in radioresistant (KYSE30R, KYSE180R) and pre-cells (KYSE30, KYSE180). Reproduced with permission [114]. Copyright 2021, Wiley-VCH GmbH. g) Western blot was used to detect the expression levels of PARP, cleaved caspase-3, γH2AX, and β-actin in KYSE30 cells after X-ray irradiation. Reproduced with permission [114]. Copyright 2021, Wiley-VCH GmbH. h) In vivo tumor growth assays to demonstrate the efficacy of nano-miR181a combined with X-ray radiotherapy. Reproduced with permission [114]. Copyright 2021, Wiley-VCH GmbH.

For instance, Ma et al. [113] fabricated a highly stable metal-carbon hybrid radiosensitizer, namely gadolinium-doped carbon dots (Gd@C-dots). Through encapsulating gadolinium ions within the carbon dots to form an interlayer structure, the leakage of gadolinium is effectively precluded, thereby minimizing the direct contact with biological tissues and reducing toxicity. Additionally, amino functionalization imparted positive charges to the surface of pPD-Gd@C-dots, facilitating the interactions with negatively charged tumor cell surfaces and enhancing the tumor retention of the hybrid radiosensitizer. Quantitative outcomes from positron emission tomography (PET) imaging indicated that the sensitizer manifested strong tumor retention in the H1299 non-small cell lung cancer model (Fig. 7a). Concurrently, uptake experiments affirmed that the high retention rate augmented the uptake of the radiosensitizer by the tumor (Fig. 7b). Furthermore, the interlayer structure guaranteed the uniform distribution of gadolinium within the tumor tissue, enhancing local radiation absorption and further boosting radiosensitivity. *In vivo* studies further attested to the superior radiosensitizing effect of this composite radiosensitizer. Compared with traditional gadolinium-based nanoparticles, this material notably improved tumor destruction (Fig. 7c).

In contrast to carbon dot-protected high-Z metals, the utilization of two-dimensional graphene (GDY) to enhance the enzyme-like activity of high-Z metals and its combination with other therapies exhibits a more potent radiosensitizing effect, especially in alleviating tumor hypoxia and overcoming radioresistance. For instance, Zhou et al. [114] fabricated a miR181a-loaded hybrid radiosensitizer, GDY-CeO_2_ (Fig. 7d), which demonstrated CAT-like activity. This hybrid nano radiosensitizer utilizes the GDY to anchor and disperse ultrasmall CeO_2_ nanoparticles with CAT-like activity, augmenting the surface area for enzymatic reactions and thereby enhancing the reaction efficiency. The enzyme reaction takes place due to the reversible conversion between Ce^4+^ and Ce^3+^ under weakly acidic tumor microenvironments or physiological conditions. Consequently, the GDY-CeO_2_ hybrid radiosensitizer displays an outstanding ability in decomposing H_2_O_2_ into O_2_, significantly alleviating tumor hypoxia (Fig. 7e), and enhancing radiation-induced DNA damage. Additionally, miR181a exerts a genetic regulatory role by directly targeting RAD17 and modulating the Chk2 pathway, increasing the susceptibility of tumor cells to X-ray-induced cell death (Fig. 7f). When loaded onto GDY-CeO_2_, this hybrid radiosensitizer integrates the dual capabilities of efficiently generating oxygen and reducing tumor cell radioresistance, leading to superior DNA damage in tumor cells under X-ray irradiation (Fig. 7g). It has consistently manifested stable therapeutic effects throughout multiple parallel experiments (Fig. 7h), rendering this hybrid material highly prospective for clinical applications.

#### 3.1.5 Metal Nanoparticle-MOF hybrid radiosensitizer for enhanced radiotherapy optimization

The metal nanoparticle-MOF hybrid radiosensitizer pertains to a composite material constituted by embedding metal nanoparticles within the framework of a metal-organic framework (MOF), which is devised to augment the efficacy of radiotherapy. Beyond the radiosensitization attributes conferred by the metal nanoparticles, this hybrid radiosensitizer exploits the MOF’s considerable surface area and porous architecture for proficient drug loading, its catalase-like activity that engenders oxygen at tumor locations, and the structural stability derived from the metal-MOF hybridization [[115], [116], [117]]. Consequently, through the combination of metal nanoparticles and MOFs, the synergistic effects are maximized, prominently enhancing radiosensitivity.

**Fig. 8:**
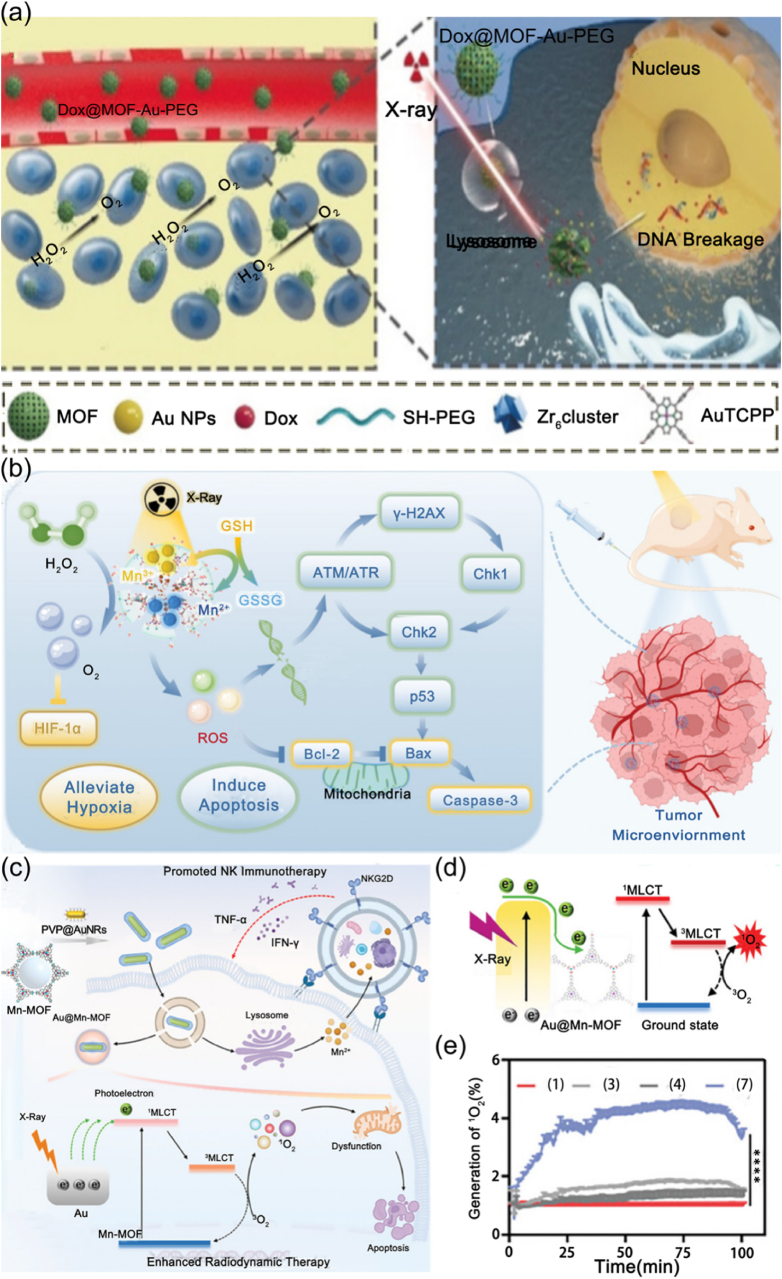
a) Schematic diagram of the main components of Dox@MOF-Au-PEG and the radiosensitization mechanism of O_2_ self-sufficiency. Reproduced with permission [118]. Copyright 2019, Wiley-VCH GmbH. b) The oxygen production capacity of FA-Mn_3_O_4_@ZIF-8 was measured using an RDPP valve oxygen fluorescence probe in the SiHa cell line. Reproduced with permission [119]. Copyright 2023, Wiley-VCH GmbH. c) Schematic of the mechanism of Au@Mn-MOF enhancement of radiokinetics. Reproduced with permission [119]. Copyright 2023, Wiley-VCH GmbH. d) Schematic representation of the mechanism of the metal-to-ligand charge transfer (MLCT) process induced by ionizing radiation. Reproduced with permission [120]. Copyright 2023, Wiley-VCH GmbH. e) DPBF was used to explore the activation of singlet oxygen under different treatments. (1) control (3) Au@Mn-MOF (4) X-ray (7) Au@Mn-MOF + X-ray. Reproduced with permission [120]. Copyright 2023, Wiley-VCH GmbH.

For example, He et al. [118] fabricated a hybrid radiosensitizer, DOX@MOF-Au-PEG, which mitigates tumor hypoxia (Fig. 8a). In this hybrid radiosensitizer, the MOF is constituted by metal nodes linked to multiple organic ligands via coordination bonds, generating a crystalline network structure. Such an arrangement endows the MOF with a porous architecture, facilitating adsorption, catalysis, and other chemical reactions. The metal nodes within the MOF, such as zirconium or titanium, imitate the active sites of catalase. Owing to the potent redox properties of these metals, they can catalyze the decomposition of H_2_O_2_ into water and oxygen, thereby enabling this hybrid radiosensitizer to alleviate the hypoxic tumor microenvironment. Additionally, the porous structure of the MOF permits the loading of chemotherapeutic drugs, such as doxorubicin, allowing for controlled drug release. During radiotherapy, the synergistic effect of chemotherapy further boosts the anticancer efficacy, achieving combined chemoradiotherapy with enhanced outcomes and reduced side effects. Furthermore, by hybridizing with Au nanoparticles, the radiosensitizer reaps the benefits of the high-Z properties of gold, which enhance radiotherapy through efficient deposition of radiation energy. In summary, this hybrid radiosensitizer significantly enhances radiotherapy efficacy by elevating oxygen levels, controlling drug release, and reinforcing the stability of the nanomaterial.

In contrast to conventional MOFs, some specific MOFs, when employed as hybrid biomaterials-based radiosensitizers, exhibit higher stability, biocompatibility, and ROS generation capacity, and are readily functionalizable. These merits render them more prospective for applications in radiosensitization. For instance, Pan and colleagues [119] fabricated a radiosensitizer, FA-Mn_3_O_4_@ZIF-8, through the hybridization of Mn_3_O_4_ with the specialized MOF architecture of ZIF-8. This hybrid system effectively depletes GSH, mitigates tumor hypoxia, and generates ROS. Owing to the large surface area and porosity of ZIF-8, it adsorbs and transports reactants efficiently. The porous structure of ZIF-8, when hybridized with Mn_3_O_4_, strengthens the interaction between Mn_3_O_4_ and GSH in the tumor microenvironment, thereby enhancing the efficiency of GSH depletion. Furthermore, when Mn_3_O_4_ is encapsulated by ZIF-8, the free imidazole nitrogen in ZIF-8 functions as a nucleophile in the solvent. This interaction creates an alkaline milieu that promotes the oxidation of Mn^2+^ to Mn^3+^ within Mn_3_O_4_, resulting in an increase in oxygen vacancies (Fig. 8b). These oxygen vacancies act as catalytically active sites, facilitating the decomposition of H_2_O_2_ and giving rise to an increased generation of oxygen as well as further catalysis of ROS production. Furthermore, owing to the facile functionalization of ZIF-8, when the hybrid radiosensitizer is functionalized with folic acid (FA), it is capable of specifically targeting tumor cells that overexpress folate receptors, enhancing the precision of drug delivery to cancerous tissues. Consequently, the hybrid radiosensitizer FA-Mn_3_O_4_@ZIF-8 markedly enhances the tumor-killing effects of X-rays. In contrast to standalone radiosensitizers, this hybrid system improves the efficacy of radiotherapy more effectively.

In addition to the significant improvement of radiosensitization by certain types of MOFs, the heterostructures formed through the hybridization of metal nanoparticles with MOFs also play a crucial role in enhancing radiosensitization. For instance, Xiong et al. [120] synthesized a heterojunction radiosensitizer, Au@Mn-MOF, by self-assembly (Fig. 8c). This material integrates AuNRs with Mn-MOF to form a heterostructure that can effectively absorb high-energy X-rays, thereby enhancing the efficacy of radiotherapy. The heterostructure reduces the requisite excitation energy and significantly augments the generation of free radicals, thereby boosting the radiosensitization performance. Furthermore, the electron transfer within the heterostructure can effectively convey energy to the catalytic active sites, promoting the Mn^2+^ mediated Fenton reaction. This process further decomposes H_2_O_2_ to generate additional free radicals, enhancing the effects of chemodynamic therapy. Upon X-ray irradiation, the ionizing radiation-induced water decomposition and metal-to-ligand charge transfer (MLCT) processes are capable of generating a considerable amount of ROS, thereby further enhancing radiosensitization. The MLCT process encompasses the charge transfer between ruthenium ions (Ru) and their ligands, where the excited electrons undergo a transition from the d-orbitals of the metal center to the π∗ orbitals of the ligand. This charge-transfer excited state elevates the molecule’s reactivity, facilitating the activation of molecular oxygen and significantly augmenting ROS production (Fig. 8d). Through this mechanism, diverse types of ROS, including singlet oxygen (^1^O_2_) and hydroxyl radicals (·OH) (Fig. 8e), can be effectively generated, thus further reinforcing radiosensitization during radiotherapy.

Although metal nanoparticle-MOF hybrid biomaterials-based radiosensitizers have demonstrated promising outcomes in preclinical investigations, their clinical translation still encounters challenges. Firstly, the synthesis of metal nanoparticle-MOF hybrid materials encompasses issues associated with large-scale production, biodegradability, and long-term toxicity assessment, which might result in accumulation within the body and augment toxicity risks. Additionally, while the high surface area and porous architecture of MOF materials facilitate drug loading and reactions, they could prematurely degrade or interact with biomolecules, influencing drug release and therapeutic efficacy. High-dose applications may also cause toxicity accumulation in vital organs such as the liver and kidneys. Furthermore, although MOF functionalization enhances tumor targeting, precise drug delivery and control of side effects remain crucial challenges. Lastly, the biocompatibility and immunogenicity of these materials demand further exploration in preclinical studies. Therefore, future research should be concentrated on resolving these issues to facilitate their application in personalized radiotherapy.

### 3.2 Organic-inorganic hybrid radiosensitizer for enhanced radiotherapy

Organic-inorganic hybrid biomaterials-based radiosensitizers integrate the biocompatibility and targeting capabilities of organic components with the high radiation absorption and ROS-generating properties of inorganic materials, attaining synergistic effects that enhance tumor radiosensitivity. Through diverse hybridization strategies, such as polymer-high-Z material hybrids, fluorocarbon-polymer hybrids, and bioinorganic combinations, these materials optimize stability, targeting, and controlled release, while alleviating hypoxia and overcoming radioresistance. This section classifies hybridization into subtypes and emphasizes their mechanisms, demonstrating superior therapeutic efficacy in comparison to single-component systems, with the potential to guide future innovations in cancer radiotherapy.

#### 3.2.1 High-Z materials are combined with polymers to enhance radiotherapy

**Fig. 9:**
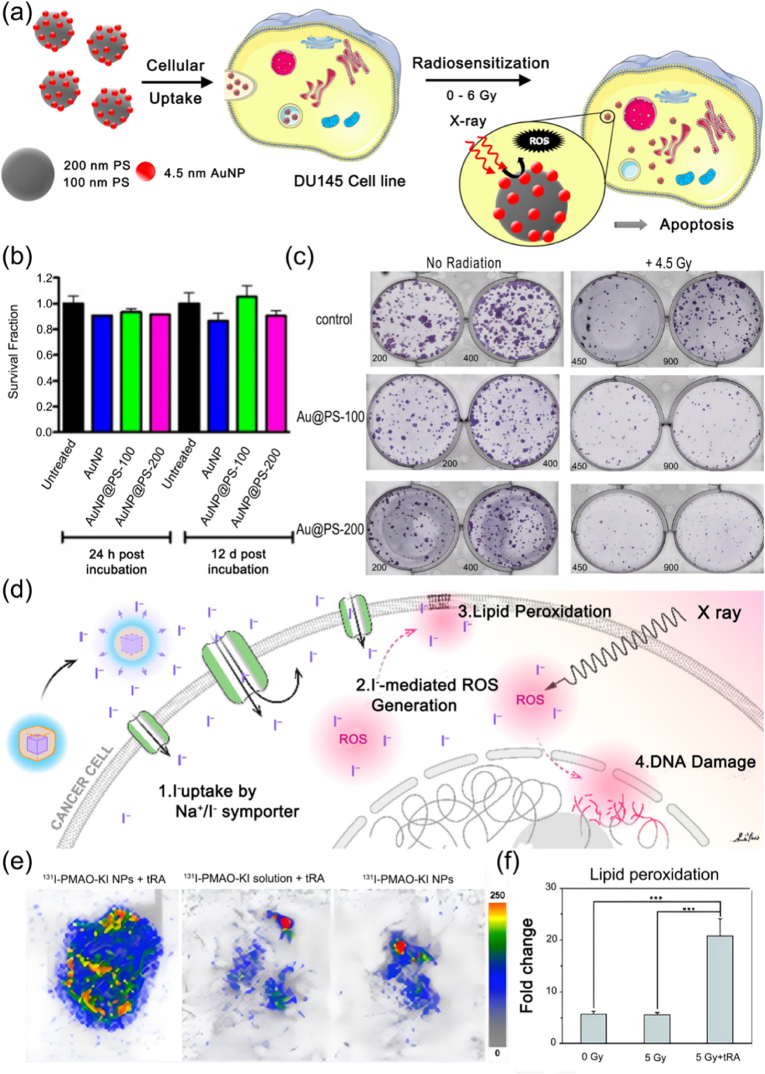
a) Schematic diagram illustrating the radiosensitization process using composite particles of AuNP@PS. Reproduced with permission [121]. Copyright 2020 American Chemical Society. b) Cell viability of DU145 cells was measured with free 4.5-nm AuNPs and AuNP@PS complex. Reproduced with permission [121]. Copyright 2020 American Chemical Society. c) The colony-forming ability of DU145 cells was compared between the control (unirradiated) and 4 Gy irradiated DU145 cells. Reproduced with permission [121]. Copyright 2021 American Chemical Society. d) Schematic representation depicting the mechanism of radiosensitization based on PMAO-KI NPs. Reproduced with permission [122]. Copyright 2021 American Chemical Society. e) Autoradiography of the tumor after treatment with various processing modalities and images are shown in pseudocolors on the right. Reproduced with permission [122]. Copyright 2021 American Chemical Society. f) Lipid peroxidation, evaluated by BODIPY 581/591 assay. Reproduced with permission [122]. Copyright 2021 American Chemical Society.

Polymer-based High-Z material hybrids radiosensitizers are composite materials that combine polymers and high-Z elements, offering excellent biological stability and cellular uptake efficiency [31], thereby outperforming single-material agents in enhancing radiotherapy efficacy. For instance, Bennie et al. [121] showed that polymer-based high-Z metal (Au-PS) hybrid biomaterials-based radiosensitizers have superior therapeutic effectiveness compared to single-material AuNPs (Fig. 9a). This hybrid radiosensitizer utilizes polystyrene (PS) as a support for AuNPs, forming a polymer-metal hybrid structure (AuNP@PS). By anchoring AuNPs on the PS surface, this structure boosts nanoparticle stability and cellular uptake efficiency. Moreover, Au-PS hybrids display low toxicity at standard concentrations, with a maximum reduction in DU145 cell viability of less than 10 % (Fig. 9b). Meanwhile, in cell-based tests, comparing the colony formation of DU145 cells between the control (non-irradiated) and 4 Gy-irradiated groups reveals that this hybrid material significantly increases cell sensitivity to radiation (Fig. 9c). Thus, polymer-high-Z hybrids exhibit remarkable radiosensitizing advantages in radiotherapy.

Unlike the hybrid biomaterials-based radiosensitizers mentioned earlier, polymer-high-Z material hybrids can further enhance the radiosensitizing performance by stabilizing and controlling the drug release, thereby optimizing the effectiveness of the radiosensitizer. For instance, Cline et al. [122] synthesized a hybrid nano-radiosensitizer, PMAO-KI NP, which exploits the efficient sodium-iodide symporter (NIS) mechanism for enhanced radiotherapy (Fig. 9d). This hybrid radiosensitizer stabilizes potassium iodide (KI) nanoparticles through a poly(maleic anhydride-alt-1-octadecene) (PMAO) coating. As a hydrophobic polymer, PMAO has limited interaction with water, reducing water penetration to the KI surface, slowing the dissolution of KI, and prolonging the release of iodide in cells. This controlled release facilitates the uptake of high-Z iodide in NIS-rich cancer cells, such as breast cancer cells, via the NIS, which operates under saturation kinetics. Additionally, tRA is employed to upregulate NIS expression, further augmenting iodide uptake in cancer cells (Fig. 9e). Once within the cell, high-Z iodide ions absorb high-energy radiation and generate secondary electrons (e.g., photoelectrons and Auger electrons). These secondary electrons induce water ionization, resulting in the production of ROS. These ROS, particularly •OH, cause substantial DNA damage, extensive lipid peroxidation (Fig. 9f), and reduced cell viability, thereby enhancing the overall radiotherapy efficacy.

In summary, although hybrid biomaterials-based radiosensitizers based on high-Z materials and polymers have demonstrated promising radiosensitizing effects in radiotherapy, their clinical transformation still encounters significant challenges. Key issues encompass precise synthesis, scalability, and insufficient long-term toxicity evaluations. Additionally, although polymer modifications can regulate drug release, achieving precise tumor targeting while minimizing side effects on normal tissues remains a major obstacle in clinical applications. Future research should concentrate on addressing the challenges of precise synthesis, toxicity assessment, targeting, and drug delivery, while enhancing the stability and biocompatibility of these materials and conducting in-depth and long-term toxicological studies. Furthermore, integrating intelligent nano-delivery systems may further optimize targeting and control of drug release, promoting their application in personalized radiotherapy.

#### 3.2.2 Fluorocarbons are combined with polymers to enhance radiotherapy

The polymer-perfluorocarbon (PFC) hybrid radiosensitizer is a nanoparticle system formed through the combination of PFCs and polymers to enhance the effectiveness of tumor radiotherapy. This hybrid sensitizer takes advantage of the high oxygen solubility and biological inertness of PFCs to effectively alleviate hypoxia within the tumor microenvironment [[123], [124], [125]], while the targeted delivery and stability provided by the polymer further optimize the oxygen release efficiency [[126], [127], [128], [129]]. As a result, the synergistic effects of PFCs and polymers offer significant advantages to this nanoplatform in radiosensitization.

**Fig. 10:**
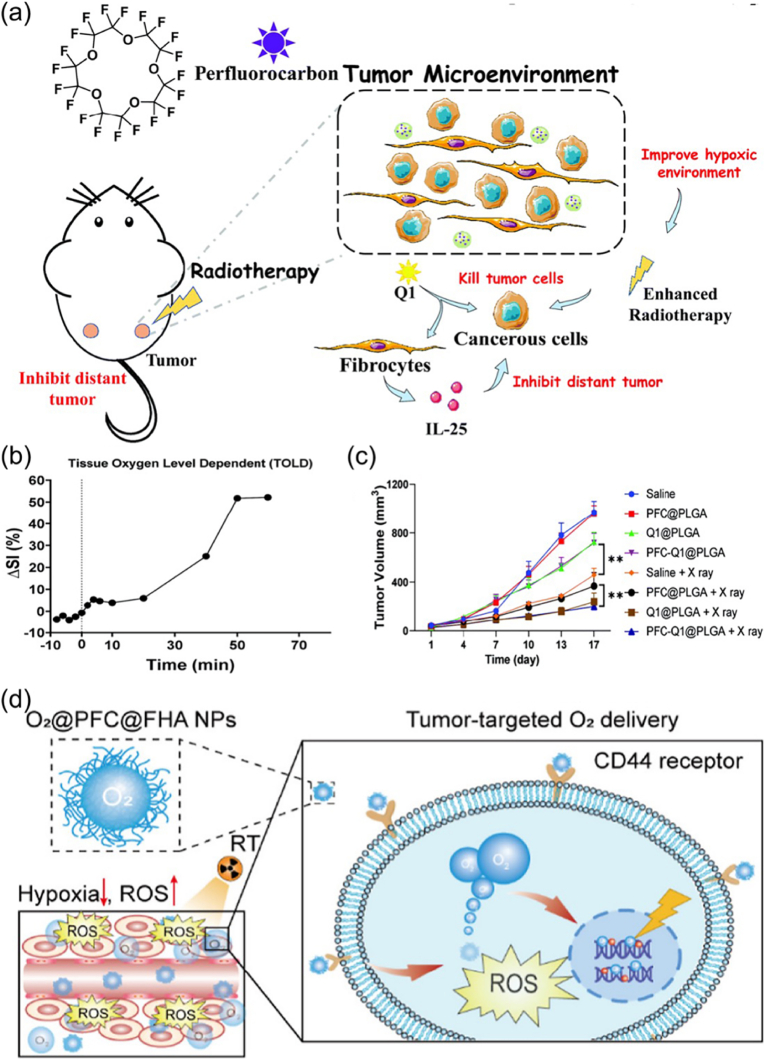
a) Schematic representation of the preparation of PLGA-based co-delivery nanoplatforms by Q1 and PFC, along with its mechanism for tumor radiotherapy treatment. Reproduced with permission [130]. Copyright 2021 Royal Society of Chemistry. b) Changes in MR signal intensity at the tumor site before and after administration of an oxygen-saturated solution containing PFC-Q1@PLGA (O_2_). Reproduced with permission [130]. Copyright 2021 Royal Society of Chemistry. c) Mean kinetics of tumor growth in different treatment groups after intravenous injection. Reproduced with permission [130]. Copyright 2021 Royal Society of Chemistry. d) Schematic illustration depicting the O_2_@PFC@FHA NP-mediated strategy for targeted delivery of oxygen to enhance anti-tumor radiotherapy. Reproduced with permission [131]. Copyright 2021 Elsevier.

For instance, Duan et al. [130] synthesized a high-oxygen-solubility hybrid radiosensitizer, PFC-Q1@PLGA, which enables stable in vivo delivery (Fig. 10a). In this hybrid radiosensitizer, the oxygen-carrying properties of PFCs provide significant benefits in enhancing radiosensitivity. Due to the high fluorine content in the molecular structure of PFCs, the distribution of their electron cloud is uniform, and the weak intermolecular forces enable them to carry a large amount of oxygen through physical adsorption (Fig. 10b). Notably, PFCs are highly chemically inert, and once hybridized with poly(lactic-co-glycolic acid) (PLGA), they show limited reactivity with other compounds in the body. Therefore, this hybrid radiosensitizer remains stable in circulation without sacrificing its oxygen-carrying capacity. Additionally, the degradation rate of PLGA can be adjusted by modifying the ratio of lactic acid to glycolic acid, enabling precise control over the drug release time in target tissues. This ensures a prolonged and sustained release within the tumor microenvironment, significantly alleviating tumor hypoxia and enhancing the effectiveness of radiotherapy. Moreover, the lignan derivative (Q1) component in this hybrid radiosensitizer is physically encapsulated within the PLGA matrix. During treatment, Q1 is gradually released at the tumor site, promoting the secretion of IL-25 by fibroblasts. High levels of IL-25 induce late-stage apoptosis in tumor cells, increasing their sensitivity to radiation therapy and further enhancing the efficacy of radiotherapy (Fig. 10c).

In contrast to the previously mentioned hybrid radiosensitizer, the polymer-PFC hybrid radiosensitizer is capable of further enhancing tumor targeting through the incorporation of a targeting polymer, thereby optimizing the efficiency of oxygen storage and delivery and making it more efficacious in tumor radiosensitization. For instance, Wang et al. [131] synthesized a fluorinated hyaluronic acid-coated perfluorocarbon hybrid radiosensitizer (O_2_@PFC@FHA NPs), endowing this hybrid radiosensitizer with an active targeting ability (Fig. 10d). Its targeted delivery capacity stems from the fact that hyaluronic acid (HA) is a natural ligand for the CD44 receptor, allowing for specific binding to CD44, which is highly expressed on the surface of tumor cells. Consequently, FHA, a fluorinated modification of HA, not only enhances the stability of nanomaterials and compatibility with perfluorocarbons but also promotes the effective accumulation of O_2_@PFC@FHA NPs at the tumor site. This enables the PFC component to alleviate the hypoxic tumor microenvironment more effectively, thereby significantly enhancing the X-ray-induced DNA damage in tumors.

Although polymer-PFC hybrid biomaterials-based radiosensitizers have shown promising radiosensitizing effects in tumor radiotherapy, their clinical translation still encounters significant challenges. Firstly, problems related to large-scale production and precise synthesis affect the stability and efficacy of the material. Secondly, although PFCs display high biological inertness, their long-term biodegradability and accumulation effects in the body require further assessment, especially regarding potential toxicity to organs such as the liver and kidneys, which might increase the risk of side effects. Additionally, targeting and precise drug delivery remain bottlenecks. Although polymer functionalization enhances targeting, optimizing the precise delivery and sustained release of the drug within the tumor microenvironment is still necessary. While fluorinated hyaluronic acid (FHA)-functionalized PFC materials target tumors via the CD44 receptor, their efficacy across different tumor types still needs validation. Therefore, further research efforts are necessary to overcome these challenges for clinical application.

#### 3.2.3 Bioinorganic hybrid biomaterials-based radiosensitizers for enhanced radiotherapy

Bioinorganic hybrid biomaterials-based radiosensitizers are composite materials integrating organic biological constituents, such as signaling peptides [132,133], macromolecular proteins [[134], [135], [136]], catalytic enzymes [137], and platelets [[138], [139], [140]], with inorganic elements like metal nanoparticles (*e.g.*, gold, platinum, and iron oxide) or metal-cluster-based MOFs. These hybrids augment the efficacy of tumor radiotherapy. By exploiting the biological functions, biocompatibility, and targeting capability of organic components, they effectively convey inorganic elements to tumor sites. This synergy enhances X-ray energy absorption, facilitates the generation of ROS, and boosts radiation-induced DNA damage.

**Fig. 11:**
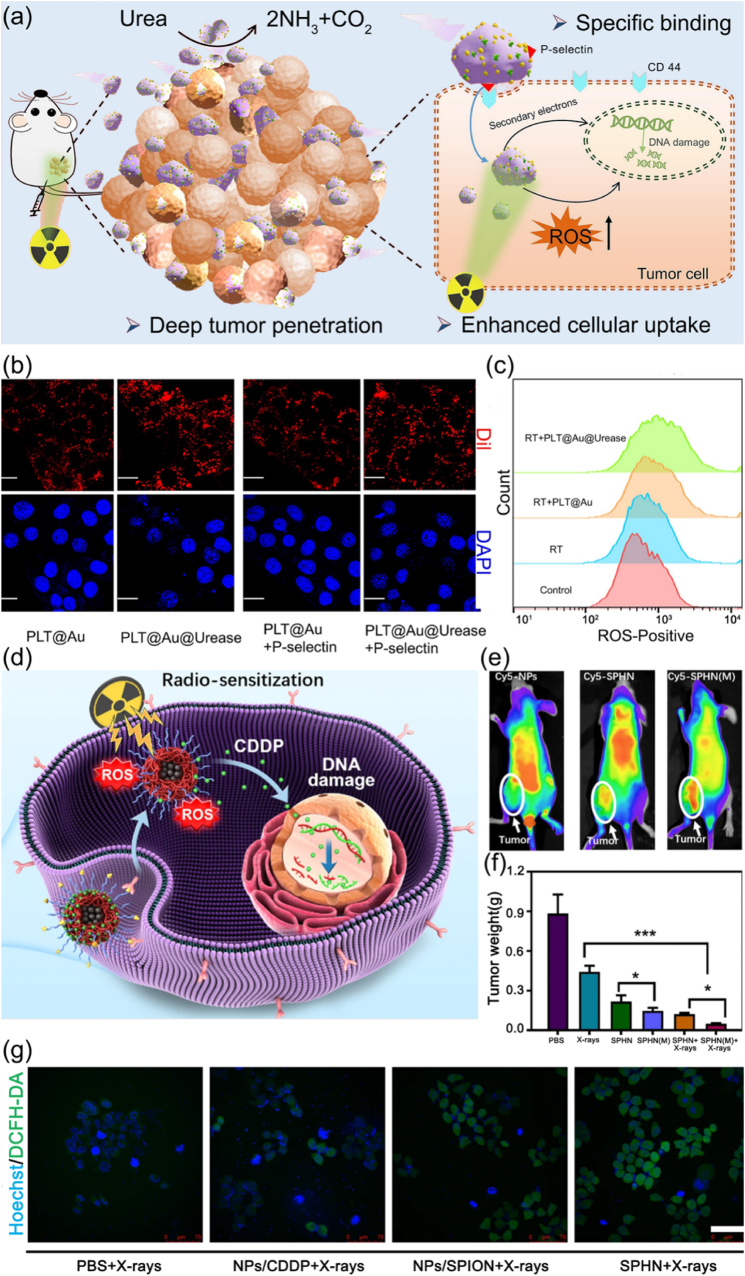
a) Schematic of the mechanism of action of PLT@Au@Urease. Reproduced with permission [141]. Copyright 2023, Elsevier. b) Laser confocal microscopy images of PLT@Au@Urease cell uptake. Reproduced with permission [141]. Copyright 2023, Elsevier. c) Flow cytometry analysis reveals intracellular ROS accumulation. Reproduced with permission [141]. Copyright 2023, Elsevier. d) Mechanistic diagram of SPHN efficiently aggregating in solid tumors through magnetic-RGD dual targeting to deliver CDDP, resulting in significantly enhanced antitumor effects. Reproduced with permission [142]. Copyright 2023, Springer Nature. e) Fluorescence imaging of uptake distribution in vivo in CNE-1 tumor-bearing mice treated with different materials. Reproduced with permission [142]. Copyright 2023, Springer Nature. f) Tumor growth curves. Reproduced with permission [142]. Copyright 2023, Springer Nature. g) The ability of CNE cells to produce ROS after treatment with different materials. Reproduced with permission [142]. Copyright 2023, Springer Nature.

Platelets, which regulate cell adhesion and migration, and catalytic enzymes, which facilitate the breakdown of metabolic byproducts within the body, play crucial biological roles. These components have been innovatively integrated to develop hybrid biomaterials-based radiosensitizers, enhancing the precision and safety of drug delivery while augmenting biocompatibility with biological tissues. For example, Liu et al. [141] developed a hybrid radiosensitizer (PLT@Au@Urease) with self-propulsion attributes (Fig. 11a). In this hybrid system, platelets (PLT) were selected as the biological carrier because of their capacity to recognize and bind to CD44 receptors on cancer cells via P-selectin on their surface. This characteristic endows PLT with the potential for tumor-targeted delivery (Fig. 11b), facilitating a more efficient utilization of the hybrid radiosensitizer. Additionally, immobilizing urease on the platelet surface confers a self-propulsion ability to PLT. This takes place as urease catalyzes the conversion of urea into ammonia and carbon dioxide, driving PLT@Au@Urease to penetrate deeper into tumor tissue and enhancing the accumulation of the radiosensitizer within the tumor. Moreover, the inorganic component of this hybrid radiosensitizer involves the in situ synthesis of AuNPs within platelets, serving as the core radiosensitizing agent. Due to their high atomic number, AuNPs effectively enhance the deposition of radiation energy within cells, thereby increasing the production of ROS (Fig. 11c), leading to more DNA double-strand breaks. Thus, through bioinorganic hybridization, PLT@Au@Urease effectively penetrates and accumulates within tumors, enhancing the radiosensitivity of cancer cells while minimizing damage to normal tissues and providing superior radiosensitizing efficacy compared to AuNPs alone.

RGD peptide is a naturally occurring functional peptide sequence within the human body. Similar to the instances mentioned earlier, its distinctive adhesive and signal-regulating attributes can be innovatively utilized in the development of hybrid biomaterials-based radiosensitizers. For example, Ding et al. [142] prepared a multifunctional superparamagnetic iron oxide nanoparticle SPION@polymer (SPHN) with enhanced radiosensitivity and dual-targeting capabilities (Fig. 11d). In this hybrid radiosensitizer, SPIONs act as the inorganic constituent, mainly enhancing tumor targeting via magnetic guidance. An external magnetic field can directly concentrate the nanosensitizer within the tumor area. Under X-ray irradiation, this hybrid radiosensitizer generates a considerable amount of ROS in the tumor microenvironment, thereby augmenting the cytotoxic effects of radiation on cancer cells. Additionally, SPIONs enable MRI guidance, enhancing therapeutic accuracy. The organic components, poly(lactic-co-glycolic acid) (PLGA) and RGD peptides, improve the biocompatibility and stability of the hybrid radiosensitizer by prolonging its circulation time in the blood. Through hybridization with SPIONs, the RGD peptides incorporated in the polymer coating selectively recognize and bind to integrin receptors overexpressed on tumor cell surfaces, achieving tumor-specific targeting (Fig. 11e). This dual-targeting mechanism enables the composite radiosensitizer to accumulate more effectively within the tumor region, resulting in more significant tumor reduction (Fig. 11f). Furthermore, the immunofluorescence detection of ROS under diverse treatment conditions affirmed that the ROS levels produced by the hybrid material SPHN within tumor cells were conspicuously higher than those generated by SPIONs alone (Fig. 11g). These outcomes manifest that the composite material provides superior radiosensitizing effects in contrast to single-component materials.

All in all, bioinorganic composite radiosensitizers have demonstrated promising radiosensitizing effects in tumor radiotherapy; however, their clinical translation encounters several challenges. The synthesis of these hybrid sensitizers is intricate, and during large-scale production, unanticipated byproducts may arise, resulting in low yields, which compromise the stability and efficacy of the material and thereby limit its clinical application. Additionally, although these materials exhibit excellent performance in vitro, their long-term usage, the toxicity of degradation products, and potential impacts on normal tissues have been insufficiently investigated, especially the systemic toxicity caused by the accumulation of metal components in the body. Furthermore, although functionalized polymers or ligands (such as RGD peptides) enhance targeting, achieving precise tumor-specific delivery while minimizing side effects on normal tissues and ensuring efficacy across various tumor types still demands further validation. Therefore, future research should be concentrated on optimizing synthesis processes, addressing challenges in large-scale production, conducting comprehensive long-term toxicity assessments, and enhancing targeting and delivery efficiency.

#### 3.2.4 Biomimetic liposome-based hybrid radiosensitizer for enhanced radiotherapy

The biomimetic liposome-based hybrid radiosensitizer described in this section integrates biomimetic liposomes with inorganic nanomaterials. This material combines liposomes with inorganic components, such as metal nanoparticles [143], enzymes, or metal alloys [144], to form an innovative radiosensitizer system. This hybrid radiosensitizer markedly enhances the efficacy of radiotherapy through multiple mechanisms, including oxygen supply, ROS enhancement, and targeted delivery, while minimizing the side effects on healthy tissues.

**Fig. 12:**
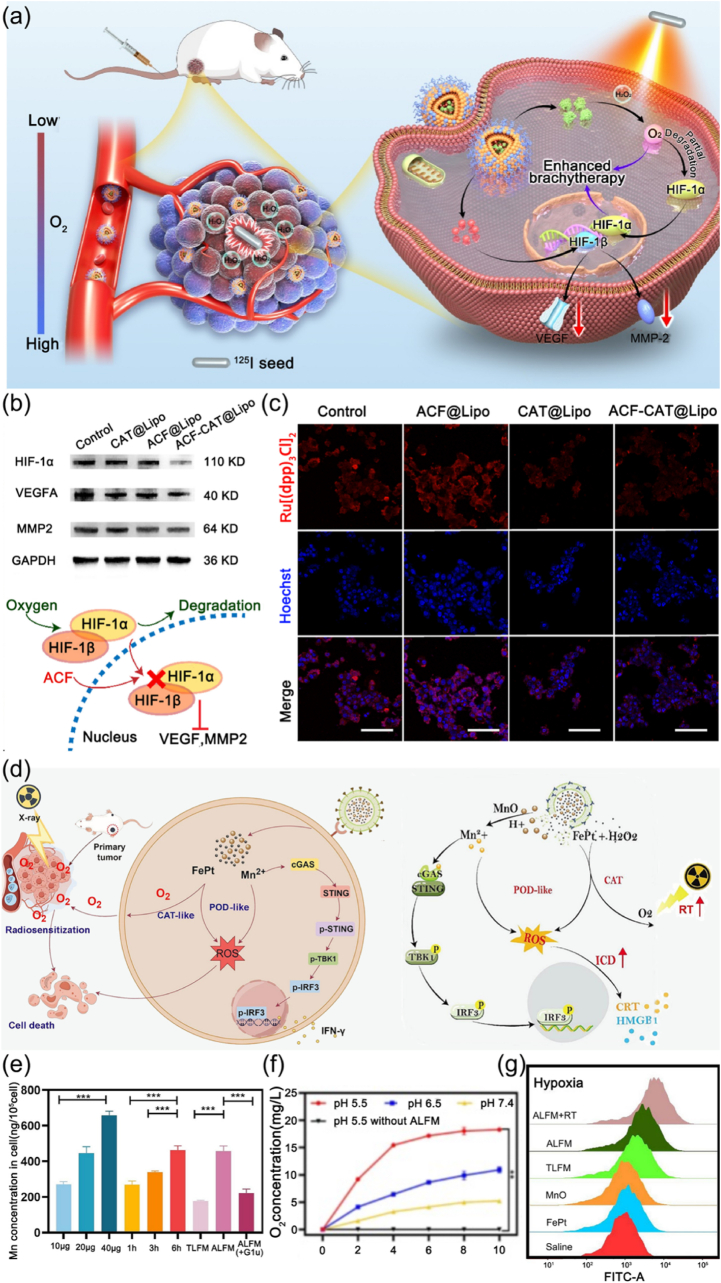
a) Schematic diagram illustrating the use of tumor oxygenating nano-liposomes and a hypoxia-inducible factor-1 inhibitor to enhance ^125^I seed brachytherapy for esophageal cancer. Reproduced with permission [145]. Copyright 2022, Elsevier. b) Western blotting images of HIF-1a, VEGFA, and MMP2 expression after different treatments under hypoxic conditions. Reproduced with permission [145]. Copyright 2022, Elsevier. c) Schematic diagram of oxygen regulation of the HIF-1 signaling pathway. Reproduced with permission [145]. Copyright 2022, Elsevier. d) CLSM images of ECA109 cells stained with [Ru(dpp)_3_Cl]_2_ probes under different treatments showed the effects of different treatments on cellular uptake, oxygenation, and ROS production. Reproduced with permission [146]. Copyright 2024, Elsevier. e) Internalization of ALFM in 4T1 cells. Reproduced with permission [146]. Copyright 2024, Elsevier. f) The O_2_ production of H_2_O_2_ treated with ALFM (100 μg/mL) at different pHs. Reproduced with permission [146]. Copyright 2024, Elsevier. g) The corresponding flow cytometry analysis for ROS production under hypoxia conditions. Reproduced with permission [146]. Copyright 2024, Elsevier.

For example, Yao et al. [145] developed a biomimetic liposome-based hybrid radiosensitizer, ACF-CAT@Lipo, which integrates a dual-function design to alleviate tumor hypoxia and overcome radiotherapy resistance, along with targeted delivery (Fig. 12a). This hybrid radiosensitizer encloses both CAT and HIF-1 inhibitor (ACF) within liposomes. CAT catalyzes the transformation of H_2_O_2_ in the tumor microenvironment into O_2_, thereby alleviating hypoxia in tumor tissues. The elevated oxygen concentration further promotes the generation of ROS during radiotherapy, thereby enhancing the cytotoxicity of radiation on tumor cells and augmenting the overall efficacy of radiotherapy. ACF, as an HIF-1α inhibitor, disrupts the function of HIF-1, suppressing the expression of hypoxia-related proteins (such as VEGF and MMP-2) (Fig. 12b and c). This contributes to reducing tumor resistance to radiotherapy, making tumor cells more vulnerable to radiation. Additionally, the organic component of this hybrid radiosensitizer employs liposomes as carriers, which can effectively enhance drug targeting and biocompatibility in tumor tissues. The utilization of liposomes also improves the stability of the radiosensitizer, ensuring that the enzyme and inhibitor exert their maximal effect at the tumor site. Consequently, by integrating CAT, ACF, and liposomes into a hybrid radiosensitizer, the synergistic effects offer enhanced tumor oxygenation, augmented ROS generation, and decreased radiotherapy resistance, leading to a more pronounced improvement in radiosensitization in contrast to individual components.

Based on the previous examples, the integration of additional strategies to overcome radiotherapy resistance and activate immune responses can further enhance radiosensitization, thereby augmenting the overall effectiveness of radiotherapy. Based on the previous examples, the integration of additional strategies to overcome radiotherapy resistance and activate immune responses can further enhance radiosensitization, improving the overall therapeutic efficacy of radiotherapy. For example, Wang et al. [146] prepared an oxygen-self-supplying hybrid radiosensitizer, ALFM, through encapsulating FePt nanoalloy and MnO nanoparticles within Astragaloside-IV-modified liposomes (Fig. 12d). The organic constituent of this hybrid system, namely the Astragaloside-IV-modified liposome, targets tumor cells by binding to the GLUT-1 receptors on their surface, facilitating the selective uptake by the tumor cells (Fig. 12e). This targeting mechanism enables ALFM to accumulate specifically in tumor tissues, enhancing drug stability and guaranteeing the maximum efficacy at the tumor site. Additionally, the inorganic components, composed of FePt nanoalloy and MnO nanoparticles, function through multiple mechanisms to further enhance radiosensitization. The high atomic number of FePt nanoalloy augments the radiation energy deposition and ROS generation within tumor cells while catalyzing the conversion of H_2_O_2_ to O_2_ (Fig. 12f), alleviating tumor hypoxia and improving the effectiveness of radiotherapy. MnO nanoparticles, via a Fenton-like reaction, convert H_2_O_2_ into ⋅OH (Fig. 12g), further enhancing ROS production and promoting oxygen generation to ameliorate the tumor microenvironment, thereby enhancing radiosensitization. The synergistic effect of the organic and inorganic components significantly elevates the tumor sensitivity to radiotherapy.

Overall, hybrid biomaterials-based radiosensitizers outperform single-material systems by integrating the targeted delivery of organic components with the catalytic effects of inorganic nanoparticles, increasing tumor accumulation, alleviating hypoxia, and promoting ROS production, thereby significantly enhancing the efficacy of radiotherapy.

### 3.3 Organic hybrid biomaterials-based radiosensitizers for enhanced radiotherapy

Organic hybrid biomaterials-based radiosensitizers combine the biocompatibility and targeting capabilities of organic components with customized functionalities to boost tumor radiosensitivity. Through strategies like dual-organic hybridization and organic nanoparticle systems, these materials facilitate precise targeting, enhanced cellular uptake, and controlled drug release. This section emphasizes various mechanisms, including redox-sensitive delivery, DNA damage amplification, and cell cycle modulation, achieved by innovative designs such as iodinated polymersomes and high-density lipoprotein-based hybrids. Compared with single-component systems, organic hybrids notably improve radiotherapy efficacy and provide valuable perspectives for the future development of radiosensitization technologies.

#### 3.3.1 Organic drug delivery systems

The organic hybrid radiosensitizer constitutes a nanoscale carrier system that integrates two distinct organic components, augmenting the radiosensitivity of tumor cells through the combination of an organic radiosensitizer and an organic nanoparticle carrier. These hybrid materials typically amalgamate the bioactivity of therapeutic agents with the targeting and controlled-release functionalities of the carrier, facilitating effective tumor targeting while minimizing adverse impacts on normal tissues.

**Fig. 13:**
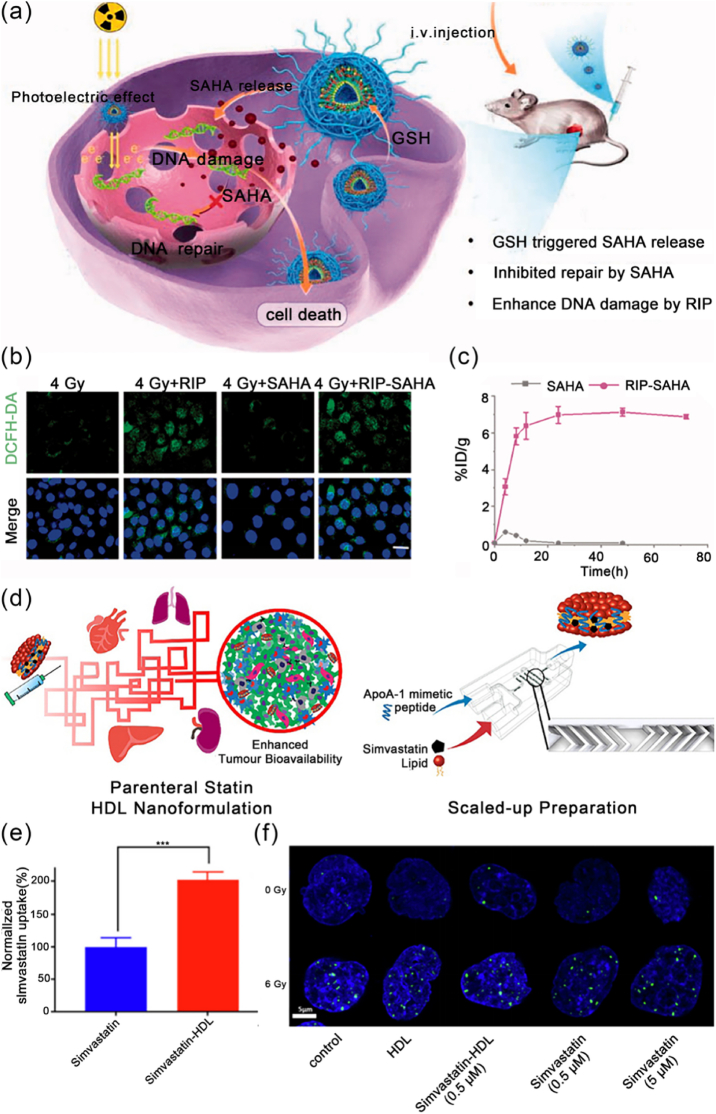
a) Schematic representation of RIP-SAHA as a bifunctional nanosensitizer. Reproduced with permission [147]. Copyright 2024, Informa Healthcare. b) Fluorescence images of ROS production in 4T1 cells after different treatments using the DCFH-DA probe. Reproduced with permission [147]. Copyright 2024, Informa Healthcare. c) Tumor uptake of ^125^I-SAHA and ^125^I-RIP-SAHA at different times was quantified by micro-SPECT/CT images. Reproduced with permission [147]. Copyright 2024, Informa Healthcare. d) To investigate the mechanism of high-density lipoprotein (HDL) nanoparticles in radiosensitization. Reproduced with permission [148]. Copyright 2022, Elsevier. e) Intracellular levels of simvastatin at t = 4 h for either simvastatin-HDL NPs or free simvastatin. Reproduced with permission [148]. Copyright 2022, Elsevier. f) DNA damage (γ-H2AX staining) 4 h after treatment. Reproduced with permission [148]. Copyright 2022, Elsevier.

For example, Zhu et al. [147] fabricated a redox-sensitive iodinated polymersome (RIP) hybridized with the histone deacetylase inhibitor (SAHA), thereby forming the hybrid radiosensitizer RIP-SAHA (Fig. 13a). In this hybrid radiosensitizer, the iodine-rich RIP component, characterized by its high atomic number, absorbs X-rays, liberating secondary electrons and generating a considerable amount of ROS (Fig. 13b). This procedure amplifies DNA damage in tumor cells, thereby potentiating the efficacy of radiotherapy. Furthermore, RIP exhibits excellent targeting and biocompatibility, allowing it to accumulate effectively in tumor sites in vivo and release SAHA within the tumor microenvironment through its redox sensitivity (Fig. 13c). Once liberated, SAHA inhibits histone deacetylase, reducing DNA-histone interactions, thereby exposing DNA to additional radiation damage and inhibiting DNA repair mechanisms in tumor cells, thereby augmenting radiosensitization. Consequently, the hybridization of these two organic components attains superior radiosensitizing effects compared to either material alone, rendering hybrid biomaterials-based radiosensitizers more efficacious than single-component alternatives.

In contrast to drug hybridization with SAHA, the combination of high-density lipoprotein (HDL) with drugs also exhibits distinct advantages in augmenting cellular internalization and enhancing radiosensitization. For example, Dehghankelishadi et al. [148]synthesized a simvastatin-based composite radiosensitizer employing HDL, designated as simvastatin-HDL (Fig. 13d). In this hybrid radiosensitizer, the HDL constituent promotes cholesterol efflux from the cell membrane, causing membrane destabilization. This instability heightens the susceptibility of tumor cells to X-rays, leading to more significant DNA damage within cancer cells. Furthermore, the HDL-simvastatin hybrid markedly boosts the cellular internalization of the composite radiosensitizer (Fig. 13e). This facilitates simvastatin’s inhibition of HMG-CoA reductase, reduces intracellular antioxidant synthesis, and induces greater oxidative stress in tumor cells. As a result, it increases radiation-induced DNA double-strand breaks and inhibits DNA repair processes, rendering tumor cells more sensitive to radiotherapy (Fig. 13f).

To offer a more lucid comprehension of the performance disparities and mechanistic benefits among diverse composite radiosensitizers, we present Table 2, Table 3. Table 2 provides a comparative summary of the performance metrics of representative composite sensitizers addressed in this section. This includes their structural features, therapeutic results, and ROS generation capacities. Table 3 further elaborates on this analysis by summarizing the efficacy, underlying mechanisms, and key physicochemical parameters of different kinds of composite radiosensitizers. Collectively, these tables systematically emphasize the synergistic effects and design advantages of hybrid materials over traditional single-component systems, thus providing valuable perspectives for future optimization and clinical translation.

**Table 2:** Performance comparison of different composite sensitizers.

Types of hybrid sensitizers	Sensitization effect	biocompatibility	stability	target	Advantages	Disadvantages	Application
Bimetallic	High	Medium	High	Medium	Enhanced radiation energy deposition; catalysis to generate O_2_ and ROS; synergy in DNA damage	Synthesis complexity; long-term biosafety concerns; limited degradability	Au-Pt [87] Ru-Cu [88]
Polymetallic	High	Medium	High	Medium	Multiple metallic properties superimposed, heterogeneous structure effect, multimodal treatment and diagnosis functions, and enhanced oxygen generation capacity	The synthesis process is highly complex and the potential toxicity increases.	Au@AgBiS_2_ [94] Au@MnS@ZnS [95]
Metal-Semiconductor	High	Medium	High	Medium	Efficient electron–hole separation; heterostructure boosts ROS; Localized Electric Field	Interface charge transfer complexity; Difficult to synthesize and reproduce; potential toxicity	CSA [101] Bi_2_S_3_@BSA-Fe_3_O_4_-FA [105] Au–Bi_2_S_3_ [107]
Metal-Carbon	Medium	High	High	Medium	Improved stability and modifiability; enzyme-mimicking behavior; controlled drug release	Potential carbon carrier degradation; complex functionalization required	Gd@C-dots [113] GDY–CeO_2_ [114]
Metal Nanoparticle-MOF	High	High	Medium	High	Porous structure for drug loading; O_2_ generation; high-Z effect; controlled release	MOF biodegradation risk; drug leakage; systemic accumulation	DOX@MOF–Au [118] FA–Mn_3_O_4_@ZIF-8 [119] Au@Mn-MOF [120]
High-Z with Polymers	High	High	Medium	High	Stable delivery; controlled release; enhances ROS via photoelectric effect	Precise control of drug release challenging; targeting specificity depends on NIS expression	Au–PS [121] PMAO–KI [122]
Fluorocarbon with Polymers	High	High	Medium	High	High O_2_ solubility; mitigates hypoxia; biocompatibility; sustained release	Long-term accumulation of PFCs; potential liver/kidney burden	PFC-Q1@PLGA [130] O_2_@PFC@FHA [131]
Bioinorganic	High	High	Medium	High	Immune-evasive targeting; self-propelling; ROS enhancement; improved tumor accumulation	Complex fabrication; risk of immune response or metal overload	PLT@Au@Urease [141] SPHNs [142]
Biomimetic Liposomes	High	High	Medium	High	Combines oxygen supply, HIF-1 inhibition, and targeting; enhanced ROS generation	Enzyme/inhibitor stability in vivo; synthesis complexity	ACF–CAT@Lipo [145] ALFM [146]
organic drugs with organic carriers	Medium	High	High	Medium	Good biocompatibility; redox/acid-triggered release; DNA repair inhibition via drugs like SAHA	Lower inherent radiosensitivity than inorganic; may require combination	RIP–SAHA [147] Simvastatin–HDL [148]

**Table 3:** Comparative analysis of composite radiosensitizers: Efficacy, mechanisms and key parameters.

Study	Hybrid Radiosensitizer	Mechanism of Action	Key Experimental Outcomes
Lin et al. [48]	Gold nanoparticles and manganese oxide (JNP Ves)	ROS generation, tumor microenvironment response (GSH)	Enhanced DNA damage, deeper tissue penetration, reduced side effects on healthy tissues
Gong et al. [57]	Calcium peroxide (CaO_2_)-HSA nanocomposite	ROS generation, oxygen release to alleviate hypoxia	Significant tumor hypoxia alleviation, enhanced radiosensitization
Weng et al. [60]	pH/hypoxia-sensitive micelles (PEG-PAA-MN)	Targeted drug delivery, pH and hypoxia responsiveness	Improved tumor targeting, enhanced radiosensitization under acidic and hypoxic conditions
Guo et al. [67]	Redox reaction between Cu^+^ and Au^3+^ to form Cu-Se-Au alloy	Stable nanoparticle loading, precise particle size control, uniform distribution	Enhanced stability, highly efficient therapeutic system, controlled nanoparticle size
Zang et al. [72]	Bi_2_WO_6_ Nanosheets	ROS generation, photocurrent response	Enhanced ROS production, strong photocurrent response, improved tumor radiotherapy efficacy
Pan et al. [80]	MGTe (GSH-modified tellurium nanoparticles coated with tumor and bacterial membranes)	ROS generation; improved biocompatibility and targeting via biomembrane fusion (tumor + bacterial membrane coating)	Enhanced ROS production; significantly improved radiosensitization; immune activation via biomimetic interface
Xia et al. [81]	Gold nanoparticle-hemoglobin composite (Au-Hb@PLT)	Oxygen-carrying capacity of hemoglobin, platelet-based targeting	Reduced tumor hypoxia, enhanced radiosensitization, better biocompatibility
Luo et al. [82]	Au-Gd-PSMA nanoparticles	MRI-guided targeting, tumor-specific targeting (PSMA)	Superior tumor targeting, minimized damage to healthy tissues, effective
Yang et al. [87]	Au-Pt bimetallic nanospheres	Radiation absorption, tumor hypoxia alleviation	Increased DNA damage, enhanced radiosensitization, reduced tumor volume
Hu et al. [88]	Ruthenium (Ru) and Copper (Cu) nanoparticles	Ru enhances energy deposition; Cu promotes ROS generation and alleviates tumor hypoxia.	Increased DNA damage, apoptosis, and ROS.Tumor volume reduction, improved radiotherapy efficacy.
Xiao et al. [94]	Au@AgBiS_2_ (gold–silver–bismuth sulfide heterostructure)	High-Z metal (Au, Bi) enhances energy deposition; Schottky barrier promotes e^−^/h^+^ separation, increases ROS generation; local electric field enhances energy delivery and DNA damage under X-rays	Significantly enhanced DNA damage and ROS levels; greater tumor suppression and survival improvement compared to single-material AuNPs, both in vitro and in vivo
Li et al. [95]	Au@MnS@ZnS (core–shell–shell heterostructure)	Au core deposits radiation energy; heterostructure enhances ROS production; paramagnetic Mn^2+^ enables MR imaging; ZnS shell stabilizes structure	Achieved effective radiosensitization and MRI contrast; enabled precise tumor imaging and improved therapeutic precision in radiotherapy
Huang et al. [101]	CSA (Copper selenide–gold nanocrystals)	Heterostructure enhances e^−^/h^+^ separation under radiation; high-Z Au increases X-ray absorption and ROS generationincreases X-ray absorption and ROS generation	Higher radiosensitization than AuNPs; greater tumor cell destruction; improved survival in mice
Nosrati et al. [105]	Bi_2_S_3_@BSA-Fe_3_O_4_-FA (Janus heterojunction nanoparticle)	Heterojunction facilitates charge separation and boosts ROS; high-Z Bi_2_S_3_ enhances radiation absorption; Fe_3_O_4_ enables Fenton reaction-based chemodynamic therapy	Significant enhancement in radiosensitization; synergistic tumor cell destruction
Wang et al. [107]	Au–Bi_2_S_3_ HNSC (Schottky-type heterostructure)	Built-in electric field at metal–semiconductor interface promotes e^−^/h^+^ separation; enhances ROS generation and DNA damage; Au improves X-ray energy deposition at tumor site	Significant DNA damage in tumor cells; superior radiosensitization efficiency
Ma et al. [113]	Gadolinium-doped carbon dots (Gd@C-dots)	High-Z metal absorption, enhanced radiation	Strong tumor retention, improved tumor destruction, enhanced
Zhou et al. [114]	GDY-CeO_2_ hybrid	Enzyme-like activity, oxygen generation, gene regulation	Alleviated tumor hypoxia, increased ROS production, enhanced DNA damage, improved radiosensitization
He et al. [118]	DOX@MOF-Au-PEG	MOF catalyzes H_2_O_2_ into O_2_ (mimicking catalase), alleviating hypoxia; porous MOF enables drug loading and controlled release; Au improves X-ray absorption	Enhanced RT efficacy through hypoxia relief, sustained DOX release, and energy deposition; improved therapeutic outcome and material stability
Pan et al. [119]	FA-Mn_3_O_4_@ZIF-8	ZIF-8 improves GSH depletion and oxygen generation via Mn^2+^ → Mn^3+^ oxidation; folic acid enhances targeting; porous structure promotes ROS production through catalysis	Improved tumor targeting, ROS generation, and oxygen delivery; markedly enhanced RT effects compared to single components
Xiong et al. [120]	Au@Mn-MOF	Heterostructure facilitates e^−^ transfer and reduces excitation energy; enhances Mn^2+^-mediated Fenton reaction; MLCT process augments ROS including ^1^O_2_ and ·OH	Synergistic RT and chemodynamic therapy; efficient ROS generation; amplified cancer cell destruction under X-ray irradiation
Bennie et al. [121]	AuNP@PS (gold nanoparticles on polystyrene)	PS stabilizes AuNPs and improves cellular uptake; Au enhances radiation absorption and ROS generation; polymer-metal hybrid shows low cytotoxicity	Increased radiosensitivity in DU145 cells; minimal toxicity at standard doses; significant reduction in colony formation after 4 Gy irradiation
Cline et al. [122]	PMAO-KI NP (polymer-coated potassium iodide)	PMAO coating slows KI dissolution, prolonging iodide release; iodide uptake via NIS transporter; iodide absorbs radiation and generates secondary electrons → ROS → DNA damage and lipid peroxidation	Upregulated NIS expression enhances iodide accumulation; elevated ROS (•OH) leads to greater DNA and membrane damage; increased radiotherapeutic efficacy in NIS-rich tumor cells
Duan et al. [130]	PFC-Q1@PLGA	PFC physically adsorbs oxygen; PLGA provides stability and controlled stability and controlled release; Q1 induces IL-25 secretion promoting apoptosis; sustained O_2_ release alleviates hypoxia and enhances radiosensitivity	Enhanced RT efficacy via dual mechanisms (oxygenation + immunostimulation); stable in vivo delivery; effective tumor microenvironment modulation
Wang et al. [131]	O_2_@PFC@FHA NPs (fluorinated HA-coated PFC)	FHA targets CD44 on tumor cells, enhancing tumor accumulation; PFC carries and releases O_2_ to alleviate hypoxia; synergy with X-ray leads to DNA damage	Significantly improved tumor-specific oxygen delivery; enhanced radiosensitization; promising platform for precise RT despite remaining translational challenges (e.g., biodegradability)
Liu et al. [141]	PLT@Au@Urease	Platelet-based delivery system carrying AuNPs and urease. PLT targets tumor via CD44 binding. Urease catalyzes urea into NH_3_ and CO_2_, driving self-propulsion. AuNPs enhance radiation deposition and ROS generation.	Enhanced tumor penetration and accumulation. Superior radiosensitization compared to AuNPs alone with minimal damage to normal tissues.
Ding et al. [142]	SPHN (SPION@polymer)	SPIONs enable magnetic targeting and MRI guidance; PLGA and RGD peptides improve biocompatibility and tumor-specific targeting. X-ray irradiation induces high ROS production.	Increased tumor accumulation and ROS production. Greater tumor inhibition and radiosensitivity than SPIONs alone.
Yao et al. [145]	ACF-CAT@Lipo	CAT converts H_2_O_2_ to O_2_, relieving hypoxia; ACF inhibits HIF-1α, reducing VEGF and MMP-2 expression; liposome enhances targeting and stability	Enhanced ROS production, reduced tumor resistance to RT, improved drug delivery and stability
Wang et al. [146]	ALFM (FePt & MnO in Astragaloside-IV-modified liposomes)	FePt enhances radiation energy deposition and catalyzes O_2_ generation; MnO generates •OH via Fenton-like reaction; liposome targets GLUT-1 on tumor cells	Increased radiation-induced DNA damage, improved tumor oxygenation, enhanced tumor-specific accumulation
Zhu et al. [147]	RIP-SAHA (Redox-sensitive iodinated polymersome with SAHA)	RIP absorbs X-rays due to iodine content, producing ROS; redox sensitivity triggers SAHA release in tumor; SAHA inhibits histone deacetylase, reducing DNA-histone interaction, increasing DNA vulnerability and inhibiting repair.	Increased DNA damage and ROS production in tumor cells; improved targeting and accumulation at tumor site; enhanced radiosensitization compared to single-component materials.
Dehghankelishadi et al. [148]	Simvastatin-HDL	HDL facilitates cholesterol efflux, destabilizing cell membrane; membrane destabilization sensitizes cells to X-rays; simvastatin enhances this effect.	Enhanced radiosensitivity via increased membrane vulnerability; improved cellular uptake and internalization; synergistic effect of HDL and simvastatin improves radiotherapy outcomes.

## 4 Summary and prospect

This Review offers a meticulous and comprehensive account of the swift advancement and extensive utilization of nascent hybrid radiotherapy-sensitizing materials within the biomedical domain, laying a robust foundation for augmenting the therapeutic efficacy of radiotherapy through the design of hybrid biomaterials-based radiosensitizers. Specifically, we undertake a summary and review of the preparation approaches and mechanisms of action of hybrid biomaterials-based radiosensitizers. The preparation methods are classified into three types in accordance with their mechanisms and processes: self-assembly, chemical synthesis, and biosynthesis. The mechanisms of action are categorized into three groups based on the physical attributes of single-component materials: inorganic hybrids, organic-inorganic hybrids, and organic hybrids. Moreover, we delve deeply into the mechanisms through which hybrid biomaterials-based radiosensitizers enhance radiotherapy, highlighting their applications and summarizing their respective advantages and limitations. Despite the advent of innovative strategies and benefits affiliated with hybrid nanomaterials for the enhancement of radiotherapy, a multitude of challenges and unresolved matters still await resolution.

Although hybrid biomaterials-based radiosensitizers have demonstrated great potential in preclinical investigations, their clinical translation remains in the initial phase. When compared with existing radiosensitizers, such as gold nanoparticles and platinum-based medications, hybrid systems present several potential benefits. These encompass improved tumor targeting, enhanced radiosensitization effects, and the capacity to integrate radiotherapy with drug delivery or immunotherapy, thereby establishing a more comprehensive treatment strategy.

Hybrid materials have the potential to overcome the key limitations of current radiosensitizers, including poor tumor specificity and toxicity to healthy tissues. By integrating the high-Z-value of metals such as gold or hafnium with organic molecules, hybrid systems not only enhance radiation absorption but also exhibit better biocompatibility. This could potentially reduce side effects when compared to traditional therapies.

Furthermore, hybrid radiosensitizers introduce a novel concept in cancer treatment, presenting multifunctional capabilities within a single system. This encompasses enhanced radiotherapy outcomes, improved targeting, and the possibility of concurrent drug delivery. These advancements have the potential to substantially enhance the effectiveness of radiotherapy, providing a promising avenue for clinical utilization.

Nevertheless, the translation of these findings into clinical practice is not without difficulties. The primary concerns involve the safety and long-term stability of these materials, along with the complexity of synthesizing hybrid systems. A significant number of these materials remain in the experimental stage, and regulatory obstacles must be surmounted before they can be implemented in clinical settings. Additionally, scaling up production while preserving the integrity of the therapeutic functions poses a major challenge. Future research should concentrate on optimizing the synthesis of these hybrid systems, enhancing their reproducibility, and ensuring their long-term safety to facilitate their clinical adoption.

Firstly, it is important to acknowledge the limitations inherent in inorganic, organic, and organic-inorganic hybrid biomaterials-based radiosensitizers. Inorganic hybrid biomaterials-based radiosensitizers, typically based on metals or other inorganic materials such as gold [[149], [150], [151]], platinum [[152], [153], [154], [155], [156]], oxides [[157], [158]] and sulfides [103,159], exert effects via physical (*e.g*., enhanced radiation deposition), and chemical (*e.g.*, ROS generation) mechanisms. However, these radiosensitizers often lack tumor specificity because inorganic materials generally lack inherent biological targeting functions, necessitating additional modifications to improve selectivity [160,161]. In contrast, organic hybrid biomaterials-based radiosensitizers, composed mainly of organic molecules or polymers, achieve sensitization through interactions with functional peptides, proteins, or cell membranes. These materials significantly enhance radiotherapy effects, including but not limited to targeted delivery, oxidative stress regulation, DNA damage amplification, and immune activation. However, organic materials typically exhibit weaker direct radiosensitization effects, and their targeting efficiency may vary depending on patient and tumor types, limiting their general applicability. Organic-inorganic hybrid biomaterials-based radiosensitizers combine the direct radiosensitization capacity of inorganic components with the targeting advantages of organic materials. Despite this theoretical advantage, practical implementation faces challenges. The metabolic and excretory difficulties of inorganic components and the susceptibility of organic components like polymers or proteins to proteolytic degradation or enzymolysis in complex biological environments constrain their application. These factors remain major bottlenecks preventing the widespread clinical use of organic-inorganic hybrid biomaterials-based radiosensitizers. To address these challenges, further optimization of targeting strategies and enhancement of radiosensitization efficiency are needed to advance their translation into practical applications. Therefore, in real-world applications, researchers should leverage the strengths of each material to design safe, effective, and controllable systems that maximize sensitization effects, focusing on surface functionalization strategies while addressing shared challenges like biocompatibility and long-term toxicity.

Secondly, the in vivo metabolism and biological safety of hybrid radiotherapy sensitizers are of critical concern. From the perspective of organic components, organic hybrid radiotherapy sensitizers typically demonstrate superior biodegradability, undergoing metabolism into small molecular products via biochemical pathways such as enzymatic or hydrolytic degradation. Nevertheless, during hepatic metabolism, the introduction of metabolizable groups like hydroxyl (-OH), carboxyl (-COOH), or thiol (-SH) to enhance hydrophilicity and facilitate excretion might lead to metabolic byproducts with potential toxicity [162]. For inorganic components, chemical modifications can prolong the circulation time of nanomaterials and lower their metabolic rates, enabling them to exert therapeutic effects for an extended period. Nevertheless, these characteristics also bring about potential health hazards, such as the long-term retention and accumulation of heavy metals in the body, which heighten toxicity and adverse side effects. An important aspect to consider is that organic-inorganic hybrid biomaterials-based radiosensitizers incorporate the potential disadvantages of both organic and inorganic components. The organic components are prone to enzymatic degradation and the influence of the complex physiological environment, which might result in the deposition of inorganic components in non-target sites. Moreover, the hepatic metabolism of organic components may produce toxic byproducts, intensifying their impact on the body. As a consequence, these hybrid sensitizers encounter significant challenges in guaranteeing biological safety, as they have to deal with the dual toxicity risks and metabolic balance related to both organic and inorganic components.

Nevertheless, challenges remain in the development of effective radiosensitizers. While traditional radiosensitizers primarily achieve their effects via physical (*e.g*., enhanced radiation deposition) and chemical (*e.g*., ROS generation) mechanisms, their clinical translation is hindered by the potential toxicity of heavy metals. To address this, firstly, developing inorganic components based on rare-earth elements (*e.g.*gadolinium [[163], [164], [165]],cerium [[166], [167], [168]]) or other low-toxicity metals (*e.g.*, iron [[169], [170], [171]-], zinc [[172], [173], [174], [175]]) is crucial for alleviating heavy metal toxicity risks. Secondly, exploring the potential of non-metallic nanomaterials, such as carbon-based quantum dots [169,176] and silicon nanoparticles [177,178], and leveraging mechanisms beyond physical enhancement, including chemical reactions (e.g., reactive oxygen species generation) and interactions with cellular signaling pathways (e.g., modulation of DNA repair mechanisms [179]), can enhance efficacy while minimizing the required radiation dose. Finally, combining radiosensitizers with auxiliary therapeutic approaches, such as chemotherapy [[180], [181], [182], [183], [184]], chemodynamic therapy (CDT) [44,185,186], or immunotherapy [187,188], can induce synergy and reduce high radiation dose-induced toxicity associated with monotherapy. Collectively, component optimization, mechanism expansion, and combination therapy strategies pave the way for designing safer and more effective radiosensitizers, while addressing shared challenges like biocompatibility and long-term toxicity.

Although hybrid biomaterial-based radiosensitizers offer substantial benefits in enhancing tumor targeting and improving radiotherapy efficacy, their clinical translation remains fraught with major challenges. At present, composite systems consisting of high atomic number materials (such as gold, hafnium, and gadolinium-based materials) and functionalized organic molecules have demonstrated promising potential for applications. These systems can effectively boost radiation absorption while minimizing harm to normal tissues. Nevertheless, concerns regarding the long-term safety, biocompatibility, and scalability of these materials significantly impede their clinical use.

For instance, AGuIX® nanoparticles [[188], [189], [190], [191]], composed of a gadolinium - based inorganic core and an organic shell, have been demonstrated in multiple preclinical investigations to significantly boost the efficacy of radiotherapy. In clinical trials, the combination of AGuIX® and MRI - guided radiotherapy has been successfully utilized in the treatment of brain tumors [192], glioblastoma [193], and cervical cancer [194], indicating promising application prospects.

Nevertheless, the clinical use of AGuIX® has also brought to light crucial issues. Firstly, adverse effects such as local reactions, nausea, and mild cognitive impairment were noted when it was used in combination with radiotherapy. Secondly, the large - scale production of these nanoparticles encounters manufacturing difficulties, including intricate processes and poor batch - to - batch stability. These problems are not exclusive to AGuIX® but rather represent common challenges faced by the entire field of hybrid radiosensitizers.

Furthermore, the structural complexity of organic-inorganic hybrid systems poses challenges to the long-term stability and reproducibility of the materials. These challenges involve unstable interactions between the organic and inorganic components, material degradation caused by environmental factors, and problems related to biodegradability. Such factors may have an impact on the long-term functionality and application effectiveness of the materials.

Therefore, the clinical translation of hybrid radiosensitizers demands a systematic approach to tackle three core challenges. In the preclinical stage, it is crucial to concentrate on enhancing long-term toxicological evaluations (especially neurotoxicity), validating the mechanism of action (such as oxygen-dependent sensitization), and leveraging humanized PDX models to enhance predictability. Regarding scalable production, GMP-compliant procedures, like one-pot methods, should be developed, along with establishing key quality control criteria for particle size, sterility, and other critical aspects. Concerning clinical development strategies, it is necessary to adhere to ICH/FDA guidelines, verifying safety (Phase I), biomarkers (Phase II), and survival benefits (Phase III) in a staged manner, while establishing a collaborative mechanism among industry, academia, and healthcare to facilitate dynamic regulation. Only by surmounting these bottlenecks can the clinical application of hybrid radiosensitizers be fully achieved, offering a novel solution for precision cancer treatment.

Beyond their application in cancer treatment, hybrid radiosensitizers present exciting prospects across a wider range of medical disciplines. Although the research on hybrid radiosensitizers stemmed from the need to enhance tumor radiotherapy, their core significance extends well beyond this. The targeted delivery ability, controllable oxygen delivery function, and multimodal imaging features exhibited by these hybrid nanomaterials are creating revolutionary opportunities in a broader array of medical fields. In the cardiovascular domain, they can act as oxygen carriers to alleviate myocardial ischemia-reperfusion injury [[195], [196], [197]]; in the treatment of neurodegenerative diseases, their ability to cross the blood-brain barrier holds promise for targeted drug delivery in Alzheimer’s disease [[198], [199], [200], [201]]; and in precision imaging diagnostics, gadolinium/hafnium-based materials' bimodal imaging potential could improve early lesion detection rates [[202], [203], [204], [205], [206]].

This finding suggests a more far-reaching vision: hybrid nanomaterials are emerging as a pivotal focus for cross-disease and cross-disciplinary medical innovation. We earnestly encourage material scientists, clinicians, and bioengineers to engage in collaborative efforts to explore this highly promising domain. The aim is not solely to combat cancer but also to redefine the diagnostic and therapeutic paradigms for a spectrum of critical diseases. By leveraging the nanoscale potential of nanotechnology, let us drive this paradigm shift from tumor radiotherapy enhancement to universal precision medicine and thereby write a new chapter in human health.

## CRediT authorship contribution statement

**Jia Liu:** Writing – original draft, Software, Resources, Data curation. **Lin Zhao:** Writing – original draft, Validation, Software, Data curation. **Yang Sun:** Visualization, Software, Resources, Formal analysis, Data curation. **Qinrui Fu:** Writing – review & editing, Supervision, Project administration, Funding acquisition, Conceptualization. **Wenjing Xiao:** Writing – review & editing, Supervision, Investigation, Conceptualization.

## Declaration of competing interest

The authors declare that they have no known competing financial interests or personal relationships that could have appeared to influence the work reported in this paper.

## Data Availability

No data was used for the research described in the article.
